# Qualitative and Quantitative Analysis of Triterpene Saponins from Tea Seed Pomace (*Camellia oleifera* Abel) and Their Activities against Bacteria and Fungi

**DOI:** 10.3390/molecules19067568

**Published:** 2014-06-06

**Authors:** Xin-Fu Zhang, Shao-Lan Yang, Ying-Ying Han, Lei Zhao, Gui-Long Lu, Tao Xia, Li-Ping Gao

**Affiliations:** 1 College of Horticulture, Qingdao Agricultural University, Qingdao 266109, China; E-Mails: zxftea@163.com (X.-F.Z.); shaolanyang@126.com (S.-L.Y.); ganxiezhenshi@163.com (L.Z.); luguilong666@126.com (G.-L.L.); 2 Key Laboratory of Tea Biochemistry & Biotechnology, Ministry of Education and Ministry of Agriculture, Anhui Agricultural University, Hefei 230036, China; E-Mail: hanyyfood@163.com; 3 School of Life Sciences, Anhui Agricultural University, Hefei 230036, China

**Keywords:** tea seed pomace, saponin, LC-ESI-IT-TOF/MS, anti-fungal activity

## Abstract

A method using LC-ESI-IT-TOF/MS and LC/UV-ELSD was established to qualitatively analyze triterpene saponins obtained from the tea seed pomace (*Camellia oleifera* Abel). In addition, the quantitative analysis of oleiferasaponin A_1_ using LC/UV was developed. The purified total saponins did not exhibit any inhibitory effects at concentrations ranging from 0.1 to 10 mg/mL against the tested bacteria, except for *Staphyloccocus aureus* and *Escherichia coli*. By contrast, higher inhibitory activity was seen against the tested fungi, especially against *Bipolaris maydis*. Following treatment with an MIC value of 250 μg/mL for 24 h, the mycelial morphology was markedly shriveled in appearance or showed flattened and empty hyphae, with fractured cell walls, ruptured plasmalemma and cytoplasmic coagulation or leakage. These structural changes hindered the growth of mycelia.

## 1. Introduction

Tea seed pomace is the byproduct of oil manufacture with the seeds of *Camellia* (*C.*) *oleifera* Abel, that includes many saponins. Zhang *et al.*, isolated, purified and identified a new saponin called oleiferasaponin A_1_ (mw C_59_H_92_O_26_ determined from the [M−H]^−^ ion at *m*/*z* 1215.57975) [1]. Huang *et al.*, identified a new compound called sasanquasaoponin (C_5__8_H_92_O_26_, SQS) [2]. Also, the major component of the saponin mixture established as camelliasaponin B_1_ (C_5__8_H_89_O_26_) was reported by Kuo [3]. All the saponins found from tea seed pomace (*C.* *oleifera*) are oleanane-type triterpenoid saponins. However, few qualitative and quantitative analytical reports exist with regard to triterpenoids in tea seed pomace.

Since natural compounds, and especially triterpenoids, have shown potential novel biological activities, we have taken increasing interest in their effects and mechanisms of action. For example, studies have demonstrated that triterpenoids in *Camellia* plants showed anti-hypercholesterolemic activity [4], antioxidant activity [5], anti-hyperlipidemic activities [6], protective effects on injury of endothelial cells [7], radical scavenging effects [8], antiallergic activity [9], inhibitory effects on gastric emptying [10] and cardioprotective effects [11]. It has also been demonstrated that triterpenoids extracted from tea seed pomace (*C.* *oleifera*) were widely used as emulsifying agents [12]. Thus, the efficacy could be increased when they were used in pesticides and bactericides. If they have activities against bacteria and fungal species, the triterpenoids extracted from tea seed pomace could be potentially materials for biopesticide manufacture.

Our group has recently extracted and isolated total saponins from tea seed pomace. In this study, an HPLC-UV/ELSD/MS method was developed for the simultaneous qualitative analysis of the main saponins, and the quantitative analysis of oleiferasaponin A_1_ as has been reported previously [1]. Their activities against bacteria and fungal species were screened. The alteration of mycelial morphology and ultrastructure was further studied, and the inhibitory effects of triterpene saponins from tea seed pomace against fungi was proved.

## 2. Results and Discussion

### 2.1. TLC and HPLC Analysis of the Crude and Purified Saponins

**Figure 1:**
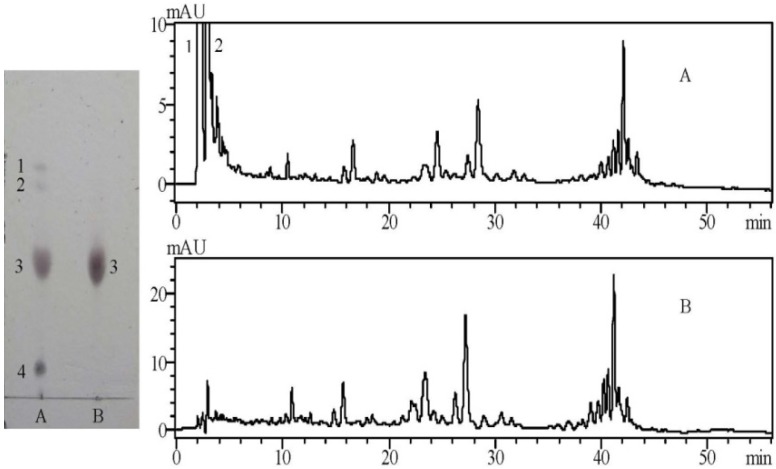
TLC and HPLC/UV analysis of saponins ((**A**) Crude saponins and (**B**) Purified saponins).

There were four clearly identified spots on the TLC plate after the crude saponins was visualized. Saponins were detected at an approximate R_f_ 0.45 and as the third spot, which was displayed as a purple-black zone, suggesting that these solids possessed the triterpenoid basic skeleton [3]. The first and second spots were inferred as flavonoids, and the fourth spot was considered sugars, which were also the major components of tea seed pomace (*C. oleifera*) [3,13]. The purified total saponins obtained by an AB-8 macroporous resin column contained highly similar triterpenoids, which appeared as a large spot due to their structure. HPLC-UV analysis at 280 nm indicated that the peaks obtained before five minutes were flavonoids because their maximum absorption wavelengths were 260 nm and 340 nm. The peaks after five minutes were inferred as triterpenoid saponins according to subsequent studies (Figure 1, Table 1). The crude saponins included two major species of flavonoids indicated as compounds **1** and **2**. By contrast, the purified total saponins rarely included flavonoids.

**Table 1:** Estimated molecular weights for saponins in tea seed pomace.

Peak	Retention Time (min)	[M−H]^−^	MS^2^	Peak	Retention Time (min)	[M−H]^−^	MS^2^
**1**	8.481	1219.6161	1081, 949	**18**	21.265	1287.6088	1119
**2**	8.987	1263.6080	-	**19**	25.252	1303.6437	-
**3**	9.303	1291.6013	1071, 891	**20**	28.290	1335.6119	1137
**4**	9.810	1201.5639	1021, 889	**21**	29.429	1379.6349	1199
**5**	10.633	1203.5867	-	**22**	30.505	1349.6264	1169, 1037
**6**	11.202	1233.5984	1053	**23**	31.138	1377.6161	1019
**7**	11.772	1203.5851	903	**24**	32.341	1347.6083	1135, 1003
**8**	12.911	1201.5714	1073	**25**	33.353	1377.6205	1181, 1001
**9**	13.544	1223.5588	1043	**26**	34.935	1349.8273	989
**10**	14.240	1217.6029	1095, 963, 783	**27**	39.556	476.2768	350
**11**	15.063	1187.5958	1035	**28**	40.128	578.3482	506
**12**	16.139	1039.5210	1007	**29**	41.264	352.9290	310
**13**	16.898	1245.6371	920	**30**	43.543	554.3477	255
**14**	17.531	1215.5797	1035, 903	**31**	45.372	595.2912	480
**15**	18.670	1317.6192	1111	**32**	46.434	580.3632	263
**16**	19.746	1287.6083	1085, 905	**33**	51.171	571.2912	470
**17**	20.506	1231.6183	1005				

Key: “-” not detected.

### 2.2. Typical Chromatograms of the Total Saponins

**Figure 2:**
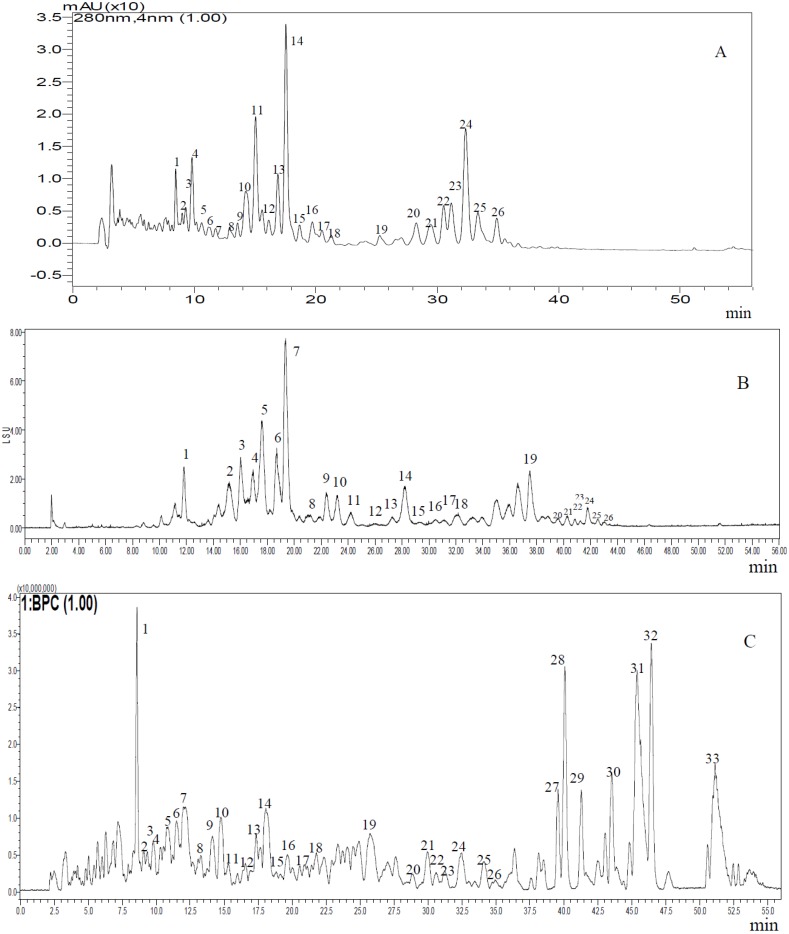
Typical chromatograms of saponins in tea seed pomace (*C. oleifera*). (**A**) Chromatogram at UV 280 nm (LC/UV); (**B**) Chromatogram at ELSD (LC/ELSD); (**C**) Total ion chromatogram (LC/TOF-MS).

Representative chromatograms of the purified total saponins are shown in Figure 2. The results indicated that triterpene saponins (compound **1** to **26**) were clearly separated and more sensitively detected at UV 280 nm (Figure 2A) than using an ELSD detector (Figure 2B). From the total ion chromatogram (Figure 2C), compounds 27 to 33 were speculated as aglycones because the molecular weights were lower, and they did not possess any ultraviolet absorption.

The qualitative determination method of saponins in tea seed pomace was built using liquid chromatography coupled with electrospray ionization hybrid ion trap and time-of-flight mass spectrometry (LC-ESI-IT-TOF/MS). The *m*/*z* ([M−H]^−^) was mainly 1,039–1,379 (Table 1), which was consistent with theasaponins A_1_ (1,189), A_2_ (1,231), A_3_ (1,273), F_1_ (1,217), F_2_ (1,259) and F_3_ (1,259) from the seeds of *Camellia sinensis* [14], assamsaponin J (1319) from tea leaves of *Camellia sinensis* var. *assamica* [10], sasanquasaponins I-V (1,232, 1,218, 1,218, 1,218, and 1,204) from the flower buds of *Camellia sasanqua* Thunb [9], yuchasaponins A (1,316), B (1,300), C (1,316) and D (1,300) from the flower buds of *C. oleifera* [15], floratheasaponins A (1,215), B (1,271), and C (1,273) from the flowers of *Camellia sinensis* [6], camellioside A (1,103), B (1,145), C (1,087), and D (1,119) from the flower buds of *Camellia japonica*. Peak 4 (Figure 2) was postulated as C_58_H_89_O_26_ with the MS/MS fragmentation patterns of the parent ion at *m*/*z* 1,201 confirming the successive loss of a hexose residue (*m*/*z* 1,021 [M-H-C_6_H_11_O_6_]^−^) and a pentose residue (*m*/*z* 889 [M-H-C_11_H_19_O_10_]^−^), which was the same as camelliasaponin B_1_ found in the seed of *Camellia japonica* L [3]. Peak 14 (Figure 2) was reported as oleiferasaponin A_1_ (*m*/*z* 1215.5797 [M−H]^−^) with the MS/MS fragmentation patterns (*m*/*z* 1,035 and 903) [1]. The preceding discussion illustrated to us that saponins found in *Camellia* have a high degree of structural identity.

**Figure 3:**
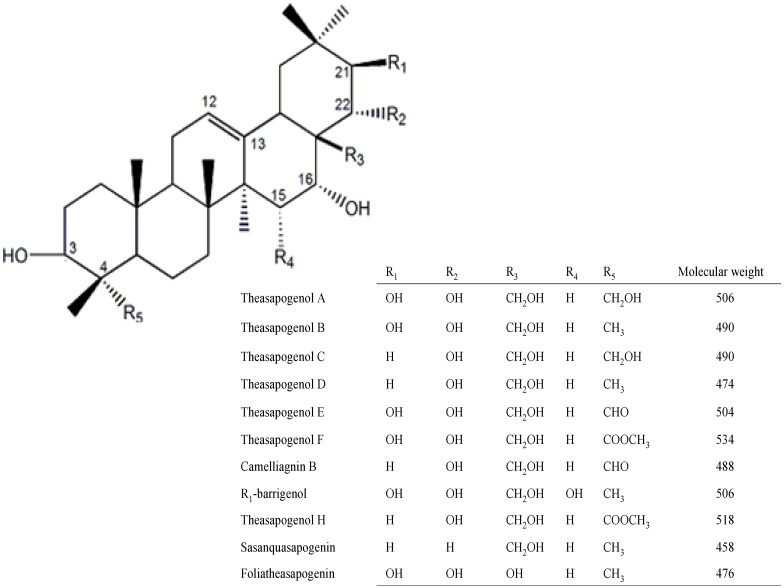
The genin structures of triterpene saponins.

It was speculated that there were many saponins in tea seed pomace with a difference in molecular weight of 2 and 30 as determined by LC-ESI-IT-TOF/MS (Table 1), which was in accordance with the structure of triterpene saponin genins (Figure 3) [6,9,10,14,16,17,18,19,20,21,22,23,24,25,26]. Compounds with a difference in molecular weight of 2 were possible because the -CHO residue replaced the -CH_2_O residue at the C4 position. Additionally, compounds with a difference in molecular weight of 30 were possible owing to the -CHO residue being replaced by the -CH_3_ residue at the C4 position and the -OH residue being replaced by the -H residue at the C21 position. On the basis of the MS^2^ data (Table 1) of [M−H]^−^ in the major compounds (**1**, **3**, **4**, **6**, **9**, **10**, **14**, **16**, **21**, **22**, **24**, **25**), we inferred *m*/*z* with a difference of 180 was due to the loss of a hexose, and a difference of 132 was due to the loss of a pentose, which also demonstrated that these compounds were triterpenoids with some glycosyls.

### 2.3. Quantitative Analysis of Oleiferasaponin A_1_

**Figure 4:**
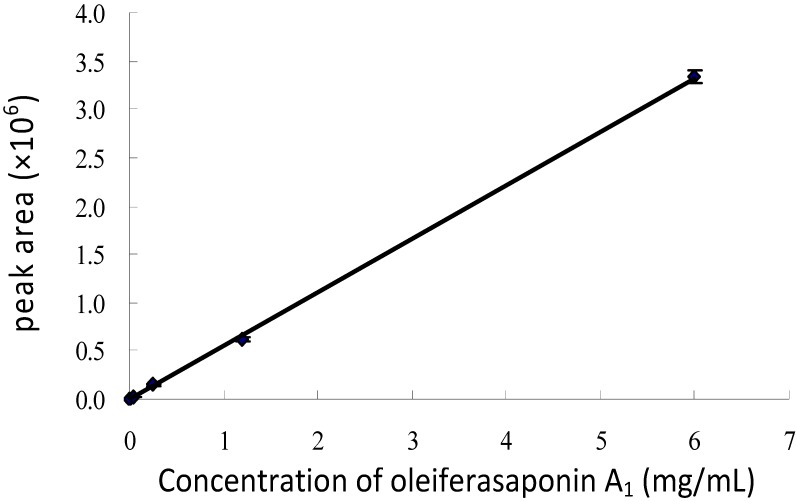
The standard curve for HPLC/UV analysis of oleiferasaponin A_1_ (280 nm).

Oleiferasaponin A_1_ was taken as the standard compound, and the standard curve for HPLC/UV analysis at a wavelength of 280 nm (Figure 4) was expressed as y = 553,272x − 2,357.1 (*R*^2^ = 0.9997), which indicated that it was a linear correlation relationship between 0.0096 and 6.0 mg/mL. And the recovery of oleiferasaponin A_1_ added into the purified total saponin at the concention 0.0096, 3.0 and 6.0 mg/mL were respectively 99.34%–105.42%, 98.20%–100.79% and 99.13%–100.26%. Most saponins in tea seed pomace have clear absorption values at 280 nm. Thus HPLC/UV analysis is a reliable and convenient method.

### 2.4. Activities against Bacteria and Fungus

The purified total saponins from tea seed pomace were examined for their anti-bacterial and anti-fungal activity (Table 2 and Table 3 respectively). The results showed that they did not exhibit any inhibitory effects at concentrations ranging from 0.1 to 10 mg/mL against the tested bacteria except for *Staphyloccocus* *aureus* and *Escherichia coli*. However, the purified total saponins at 1 mg/mL and 10 mg/mL showed low inbibitory effects against *Staphyloccocus* *aureus* and *Escherichia coli*. By contrast, purified total saponins from tea seed pomace had higher inhibitory activities against the tested fungi, especially against *B. maydis* and *Fusarium moniliforme sheld*. The purified total saponins at 0.1 mg/mL significantly inhibited the growth of mycelia. In addition, the anti-fungal effects of the purified total saponins also showed a concentration-dependent tendency. Thus the saponins from tea seed pomace could be exploited as fungal inhibitors.

**Table 2:** Anti-bacterial activity of different concentrations of saponins from tea seed pomace on nutrient agar plates incubated at 37 °C for 24 h (inhibitory zone diameter: mm).

Bacteria	Concentration of Purified Total Saponins (mg/mL)		
	0.1	1	10
*Micrococcus tetragenus*	-	-	-
*Bacillus subtilis*	-	-	-
*Pseudomonas fluorescens*	-	-	-
*Salmonella enterica*	-	-	-
*Shigellae*	-	-	-
*Staphyloccocus aureus*	-	10.0 ± 1.5 b	13.1 ± 1.6 a
*Escherichia coli*	-	-	12.2 ± 1.8

Means (*n* = 3) ± SD followed by different letters (a, b and c) indicated significantly different scores in the same bacteria, according to Duncan’s multiple range tests at *p* = 0.05 level. Key: “-” not detected.

**Table 3:** Anti-fungal activity of different concentrations of saponins from tea seed pomace on potato dextrose agar plates incubated at 27 °C for 72 h (inhibitory zone diameter: mm).

Fungi	Concentration of Purified Total Saponins (mg/mL)		
	0.1	1	10
*Bipolaris maydis*	20.1 ± 1.50 c	59.3 ± 2.31 b	67.1 ± 2.55 a
*Fusarium moniliforme sheld*	14.1 ± 1.19 c	28.8 ± 1.79 b	61.8 ± 4.86 a
*Fusarium oxysporum f.* sp. *lycopersici*	-	6.1 ± 0.35 b	39.1 ± 1.95 a
*FusaHum graminearum Sehw*	-	5.8 ± 1.05 b	23.8 ± 1.78 a
*Fusarium oxysporum f.* sp. *varsinfectum*	-	3.7 ± 0.96 b	14.1± 1.73 a
*Gloeosporium theae sinensis Miyake*	-	-	8.0 ± 1.50

Means (*n* = 3) ± SD followed by different letters (a, b and c) indicate significantly different scores in the same fungi, according to Duncan’s multiple range tests at *p* = 0.05 level. Key: “-” not detected.

### 2.5. The Alteration of Mycelial Morphology and Ultrastructure

**Figure 5:**
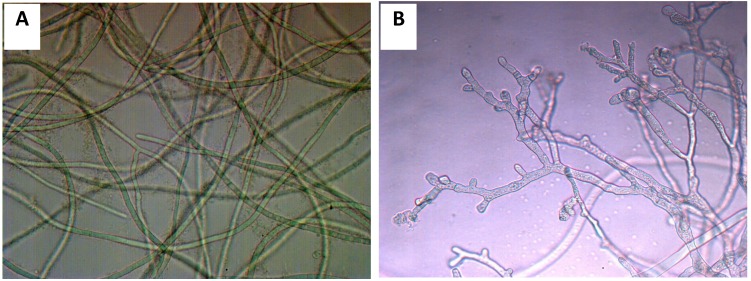
The morphological alteration of *B. maydis* mycelium treated by total saponin at a concentration of 250 μg/mL for 24 h observed with optical microscope. (**A**) Healthy mycelium; (**B**) treated mycelium.

The mycelia of *B. maydis* were tenuous and smooth (Figure 5A). Among the experimental treatments, the purified total saponins had prominent inhibitory effects against *B. maydis* with an MIC value of 250 μg/mL. Following optical microscopic observation of the mycelium of *B. maydis* treated with 250 μg/mL purified total saponins for 24 h, we discovered that mycelial morphology appeared markedly shriveled, which hindered the growth of the mycelium (Figure 5B).

**Figure 6:**
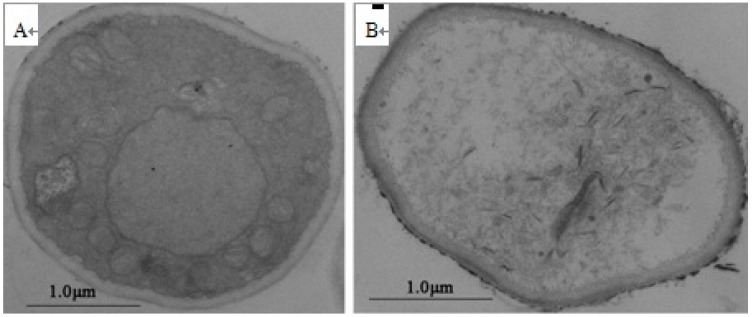
Transmission electron microscopy of mycelial ultrastructure of *B. maydis* treated by total saponin at a concentration of 250 μg/mL for 24 h. (**A**) Healthy mycelium; (**B**) treated mycelium; A ×7,000 and B ×10,000 magnification.

With transmission electron microscopy, the ultrastructure showed irreversible alterations caused by purified total saponins. We observed ruptured cell walls, plasmalemma and cytoplasmic coagulation or leakage (Figure 6B). Based on our observations, we hypothesized that purified total saponins destroyed the cell wall of *B. maydis* hyphae, damaged the cell membrane, penetrated the cytoplasm, and acted on major organelles. Subsequently, the hyphae were collapsed and appeared squashed due to a large loss in cytoplasm, and severe destruction of the organelles. Similarly, total saponins ruptured the hard cell wall structure, and act on the sporoplasm to kill the conidia. Total saponins in tea seed pomace could potentially provide a safe and environmentally friendly fungicide in the future.

## 3. Experimental Section

### 3.1. General

High-pressure liquid chromatography (HPLC) analysis was performed for the crude and purified saponins on a liquid chromatograph (Shimadzu, Kyoto, Japan) with a prominence diode array detector Model SPD-M20A. HPLC analysis was also performed for the typical chromatograms on a WATERS 600 liquid chromatograph (Waters, Milford, PA, USA) with an ultraviolet (UV) detector and an evaporative light scattering detector (ELSD). LC-ESI-IT-TOF/MS analyses were performed on a Shimadzu LCMS-IT-TOF instrument equipped with a Shimadzu Prominence HPLC system. The purification was performed using an AB-8 macroporous resin column (Bonc, Cangzhou, China), and thin layer chromatography (TLC) was performed using a GF_254_ plate (Qingdao Shenghai Silicon Material Co. Ltd., Qingdao, China). The observation of mycelial morphology of *B. maydis* was done using an optical microscope (Motic, Chengdu, China) and the observation of the mycelial structure of *B. maydis* was done using a HT7700 transmission electron microscope (Hitachi, Tokyo, Japan).

### 3.2. Plant Material

Tea seed pomace (*C. oleifera*) was collected from a factory site in Shucheng, Anhui, China, and was deposited at the Key Laboratory of Tea Biochemistry and Biotechnology, Ministry of Education and Ministry of Agriculture, Anhui Agricultural University, Hefei, China. The test microorganisms were obtained from the School of Life Sciences, Anhui Agricultural University, Hefei, China.

### 3.3. Preparation of Total Saponins

The extraction and purification of total saponins from tea seed pomace (*C. oleifera*) were performed as previously described [1]. The extraction process was done using methanol three times, following which a brown syrup was obtained, which was then purified with a nanofiltration membrane. Next, the concentrated solution was subjected to AB-8 macroporous resin column chromatography with stepwise gradients of ethanol and water (at rations of: 0:100, 30:70, 70:30, and 100:0, *v*/*v*). The third sub-fraction contained total saponins and was then subjected to HPLC analysis and tests of anti-bacterial and anti-fungal activities.

### 3.4. TLC Analysis Conditions

The crude and purified saponins were distributed with CH_2_Cl_2_:CH_3_OH:H_2_O (80:60:5, *v*/*v*/*v)* on a TLC plate. The components were visualized by spraying with 1% (*w*/*v*) Ce(SO_4_)_2_ in 10% (*v*/*v*) aqueous H_2_SO_4_ followed by heating at 120 °C.

### 3.5. Chromatographic Conditions and LC/MS Confirmation Analysis

The Shimadzu LC-20 system consisted of a DGU-20A5 pump, Prominence diode array detector, Model SPD-M20A and a 5 μL injection loop. The Waters 600 liquid chromatograph comprised a quaternary pump, a vacuum degasser, a manual sample injector, a 5 μL injection loop, a column oven, an ELSD detector and an UV detector. An Agilent Zorbax eclipse plus C18 (5 μM; 250 × 4.6 mm i.d.) column was employed, at 25 °C.

HPLC conditions were as follows: eluent A, 0.2% acetic acid in water; eluent B, acetonitrile; gradient, 0–15 min (35%–40% B), 15–45 min (40%–60% B), 45–55 min (60%–80% B), 55–56 min (80%–35% B); flow rate, 1.0 mL/min; UV detection was set at a wavelength of 280 nm; the pressure of carrier gas and temperature of the drift tube were set at 25 psi and 80 °C respectively in the HPLC-ELSD experiment.

The LCMS-IT-TOF instrument was equipped with an ESI source and the optimized MS conditions were as follows: negative ion mode; nebulizing gas (N_2_), 1.5 L/min; drying gas (N_2_) pressure, 100 kPa; curved desolvation line (CDL) temperature, 200 °C; heat block temperature, 200 °C; detector voltage, 1.7 kV; electrospray voltage, −3.5 kV; scan range, *m*/*z* 200–2,000 for MS^1^ and 200–2000 for MS^2^. TOF region pressure, 1.4 × 10^−4^ Pa; ion trap pressure, 1.8 × 10^−2^ Pa, and an ion accumulated time of 30 m; The MS^n^ data were collected in automatic mode, and the software could automatically select precursor ions for MS^n^ analysis according to criteria settings. The data acquisition and analyses were performed by LCMS Solution Version 3 software (Shimadzu) [27].

### 3.6. Standard Curve for HPLC/UV Analysis

Oleiferasaponin A_1_ from tea seed pomace (*C. oleifera*) was previously obtained, and was taken as the standard substance. The concentrations were 6, 1.2, 0.24, 0.048, 0.0096 mg/mL and the peak areas at a wavelength of 280 nm were calculated by HPLC analysis, which were performed three times. Gradient elution as described above was employed.

### 3.7. Assay of Anti-Bacterial Activity

Antibacterial activities were evaluated by using the bore-hole plate diffusion method [28,29]. The purified total saponins from tea seed pomace were dissolved in distilled water with the final concentration of 0.1, 1 and 10 mg/mL. Six mm wide holes were bored with a sterilized steel borer into the Nutrient Agar Media (beef extract 3 g, peptone 10 g, agar 17 g, NaCl 5 g, H_2_O 1,000 mL, pH 7.2) in the Petri dish that was inoculated with the test microorganism. The solution of the compounds (60 μL) at specific concentrations were added into each of the holes and distilled water was used as the control group. After the plates were incubated at 37 °C for 24 h, the diameters of the inhibition zones were measured and recorded. The assays were performed three times in order to guarantee reproducibility of results.

### 3.8. Assay of Antifungal Activity

The antifungal activity assay the against plant pathogenic fungi was carried out on 100 mm × 15 mm Petri-dishes each containing 10 mL potato dextrose agar (PDA) (200 g of potato infusion, 20 g of glucose, and 20 g of agar in 1 L of distilled water) [3]. After the mycelial colony had developed, sterile Oxford cups (6 mm in diameter) were placed at a distance of 5 mm away from the rim of the mycelial colony. Next, 60 μL of the tested sample was added to each of the oxford cups. The Petri dishes were incubated at 27 °C until the fungi had crossed the Oxford cup containing the distilled water as the control, whereas the mycelial growth of the fungi had formed inhibition zones around the Oxford cups containing samples with anti-fungal activity [30]. The mycelia of the fungi were observed by optical microscopy, and the observation of the mycelial structure was achieved by visual analysis with a transmission electron microscopy as previously reported [31,32].

## 4. Conclusions

The qualitative analytical method of analyzing triterpene saponins from the tea seed pomace (*C. oleifera*) was developed with LC-ESI-IT-TOF/MS and LC/UV-ELSD. Twenty-six major triterpenoids in the tea seed pomace were identified. HPLC/UV analysis at a wavelength of 280 nm was a reliable and convenient quantitative method for analyzing oleiferasaponin A_1_.

Purified total saponins did not exhibit any inhibitory effects at the concentrations ranging from 0.1 to 10 mg/mL against tested bacteria, with the exception of *Staphyloccocus aureus* and *Escherichia coli*, whereas they displayed higher inhibitory activities against tested fungi, and especially against *B. maydis*. Observations using optical microscopy and transmission electron microscopy revealed that following treatment with an MIC of 250 μg/mL for 24 h, the morphology of the mycelia were markedly shriveled or appeared as flattened and empty hyphae, with fractured cell walls, ruptured plasmalemma and cytoplasmic coagulation or leakage, which hindered the growth of the mycelia. Thus, it is possible for triterpene saponins found in the tea seed pomace to provide a potential of being a safe and environmentally friendly fungicide in future applications.

## Supplementary Materials

Supplementary materials can be accessed at: http://www.mdpi.com/1420-3049/19/6/7568/s1.

## References

[1] Zhang X.F., Han Y.Y., Bao G.-H., Ling T.J., Zhang L., Gao L.P., Xia T. (2012). A new saponin from tea seed pomace *(Camellia oleifera* Abel) and its protective effect on PC12 Cells. Molecules.

[2] Huang H.Q., Zhang X., Xu Z.X., Su J., Yan S.K., Zhang W.D. (2009). Fast determination of saikosaponins in Bupleurum by rapid resolution liquid chromatography with evaporative light scattering detection. J. Pharm. Biomed..

[3] Kuo P.C., Lin T.C., Yang C.W., Lin C.L., Chen G.F., Huang J.W. (2010). Bioactive saponin from tea seed pomace with inhibitory effects against *Rhizoctonia solani*. J. Agric. Food Chem..

[4] Matsui Y., Kobayashi K., Masuda H., Kigoshi H., Akao M., Sakurai H., Kumagai H. (2009). Quantitative analysis of saponins in a tea-leaf extract and their antihypercholesterolemic activity. Biosci. Biotechnol. Biochem..

[5] Hu J.L., Nie S.P., Huang D.F., Li C., Xie M.Y. (2012). Extraction of saponin from *Camellia oleifera* cake and evaluation of its antioxidant activity. Int. J. Food Sci. Technol..

[6] Yoshikawa M., Morikawa T., Yamamoto K., Kato Y., Nagatomo A., Matsuda H. (2005). Floratheasaponins A-C, acylated oleanane-type triterpene oligoglycosides with anti-hyperlipidemic activities from flowers of the tea plant (*Camellia sinensis*). J. Nat. Prod..

[7] Huang Q., He M., Chen H., Shao L., Liu D., Luo Y., Dai Y. (2007). Protective effects of sasanquasaponin on injury of endothelial cells induced by anoxia and reoxygenation* in vitro*. Basic Clin. Pharmacol. Toxicol..

[8] Sugimoto S., Yoshikawa M., Nakamura S., Matsuda H. (2009). Medicinal Flowers. Xxv. Structures of Floratheasaponin J and Chakanoside Ii from Japanese Tea Flower, Flower Buds of *Camellia Sinensis*. Heterocycles.

[9] Matsuda H., Nakamura S., Fujimoto K., Moriuchi R., Kimura Y., Ikoma N., Hata Y., Muraoka O., Yoshikawa M. (2010). Medicinal Flowers. XXXI. Acylated Oleanane-Type Triterpene Saponins, Sasanquasaponins I-V, with Antiallergic Activity from the Flower Buds of Camellia sasanqua. Chem. Pharm. Bull..

[10] Murakami T., Nakamura J., Kageura T., Matsuda H., Yoshikawa M. (2000). Bioactive saponins and glycosides. XVII. Inhibitory effect on gastric emptying and accelerating effect on gastrointestinal transit of tea saponins: Structures of assamsaponins F, G, H, I, and J from the seeds and leaves of the tea plant. Chem. Pharm. Bull..

[11] Liao Z., Yin D., Wang W., Zeng G., Liu D., Chen H., Huang Q., He M. (2009). Cardioprotective effect of sasanquasaponin preconditioning via bradykinin—NO pathway in isolated rat heart. Phytother. Res..

[12] Chen Y.F., Yang C.H., Chang M.S., Ciou Y.P., Huang Y.C. (2010). Foam properties and detergent abilities of the saponins from *Camellia oleifera*. Molecules.

[13] Chaicharoenpong C., Petsom A. (2009). Quantitative thin layer chromatographic analysis of the saponins in tea seed meal. Phytochem. Anal..

[14] Morikawa T., Li N., Nagatomo A., Matsuda H., Li X., Yoshikawa M. (2006). Triterpene saponins with gastroprotective effects from tea seed (the seeds of *Camellia sinensis*). J. Nat. Prod..

[15] Sugimoto S., Chi G., Kato Y., Nakamura S., Matsuda H., Yoshikawa M. (2009). Medicinal Flowers. XXVI. structures of acylated oleanane-type triterpene oligoglycosides, yuchasaponins A, B, C, and D, from the flower buds of Camellia oleifera-gastroprotective, aldose reductase inhibitory, and radical scavenging effects. Chem. Pharm. Bull..

[16] Yoshikawa M., Morikawa T., Nakamura S., Li N., Li X., Matsuda H. (2007). Bioactive saponins and glycosides. XXV. Acylated oleanane-type triterpene saponins from the seeds of tea plant (*Camellia sinensis*). Chem. Pharm. Bull..

[17] Yoshikawa M., Murakami T., Yoshizumi S., Murakami N., Yamahara J., Matsuda H. (1996). Bioactive saponins and glycosides. 5. Acylated polyhydroxyolean-12-ene triterpene oligoglycosides, camelliasaponins A(1), A(2), B-1, B-2, C-1, and C-2, from the seeds of Camellia japonica L: Structures and inhibitory activity on alcohol absorption. Chem. Pharm. Bull..

[18] Yoshikawa M., Morikawa T., Li N., Nagatomo A., Li X., Matsuda H. (2005). Bioactive saponins and glycosides. XXIII. Triterpene saponins with gastroprotective effect from the seeds of *Camellia sinensis*—Theasaponins E-3, E-4, E-5, E-6, and E-7. Chem. Pharm. Bull..

[19] Yoshikawa M., Nakamura S., Kato Y., Matsuhira K., Matsuda H. (2007). Medicinal flowers. XIV. New acylated oleanane-type triterpene oligoglycosides with antiallergic activity from flower buds of Chinese tea plant (*Camellia sinensis*). Chem. Pharm. Bull..

[20] Kitagawa I., Hori K., Motozawa T., Murakami T., Yoshikawa M. (1998). Structures of new acylated oleanene-type triterpene oligoglycosides, theasaponins E1 and E2, from the seeds of tea plant, *Camellia sinensis* (L.) O. Kuntze. Chem. Pharm. Bull..

[21] Murakami T., Nakamura J., Matsuda H., Yoshikawa M. (1999). Bioactive saponins and glycosides. XV. Saponin constituents with gastroprotective effect from the seeds of tea plant, *Camellia sinensis* L. var. assamica Pierre, cultivated in Sri Lanka: Structures of assamsaponins A, B, C, D, and E. Chem. Pharm. Bull. (Tokyo).

[22] Kobayashi K., Teruya T., Suenaga K., Matsui Y., Masuda H., Kigoshi H. (2006). Isotheasaponins B1-B3 from *Camellia sinensis* var. sinensis tea leaves. Phytochemistry.

[23] Morikawa T., Nakamura S., Kato Y., Muraoka O., Matsuda H., Yoshikawa M. (2007). Bioactive saponins and glycosides. XXVIII. New triterpene saponins, foliatheasaponins I, II, III, IV, and V, from Tencha (the leaves of *Camellia sinensis*). Chem. Pharm. Bull..

[24] Lu Y., Umeda T., Yagi A., Sakata K., Chaudhuri T., Ganguly D.K., Sarma S. (2000). Triterpenoid saponins from the roots of tea plant (*Camellia sinensis* var. assamica). Phytochemistry.

[25] Akagi M., Fukuishi N., Kan T., Sagesaka Y.M., Akagi R. (1997). Anti-allergic effect of tea-leaf saponin (TLS) from tea leaves (*Camellia sinensis* var. sinensis). Biol. Pharm. Bull..

[26] Yoshikawa M., Wang T., Sugimoto S., Nakamura S., Nagatomo A., Matsuda H., Harima S. (2008). Functional saponins in tea flower (flower buds of *Camellia sinensis*): Gastroprotective and hypoglycemic effects of floratheasaponins and qualitative and quantitative analysis using HPLC. J. Pharm. Soc. Jpn..

[27] Guo X.Y., Han J., Ye M., Ma X.C., Shen X., Xue B.B., Che Q.M. (2012). Identification of major compounds in rat bile after oral administration of total triterpenoids of *Ganoderma lucidum* by high-performance liquid chromatography with electrospray ionization tandem mass spectrometry. J. Pharm. Biomed..

[28] Feng N., Ye W., Wu P., Huang Y., Xie H., Wei X. (2007). Two new antifungal alkaloids produced by Streptoverticillium morookaense. J. Antibiot..

[29] Ling T.J., Ling W.W., Chen Y.J., Wan X.C., Xia T., Du X.F., Zhang Z.Z. (2010). Antiseptic activity and phenolic constituents of the aerial parts of Vitex negundo var. cannabifolia. Molecules.

[30] Lam Y.W., Wang H.X., Ng T.B. (2000). A robust cysteine-deficient chitinase-like antifungal protein from inner shoots of the edible chive Allium tuberosum. Biochem. Biophys. Res. Commun..

[31] Shao X., Cheng S., Wang H., Yu D., Mungai C. (2013). The possible mechanism of antifungal action of tea tree oil on Botrytis cinerea. J. Appl. Microbiol..

[32] Li W.R., Shi Q.S., Ouyang Y.S., Chen Y.B., Duan S.S. (2013). Antifungal effects of citronella oil against Aspergillus niger ATCC 16404. Appl. Microbiol. Biotechnol..

