# Supplementary material for: Qualitative and Quantitative Analysis of Triterpene Saponins from Tea Seed Pomace (Camellia oleifera Abel) and Their Activities against Bacteria and Fungi
Source: Molecules. 2014 Jun 6;19(6):7568–80. doi: 10.3390/molecules19067568 (PMC6271494; doi:10.3390/molecules19067568)

Supplementary

MS spectrum results for Table 1

Peak 1   Retention time (min): 8.481

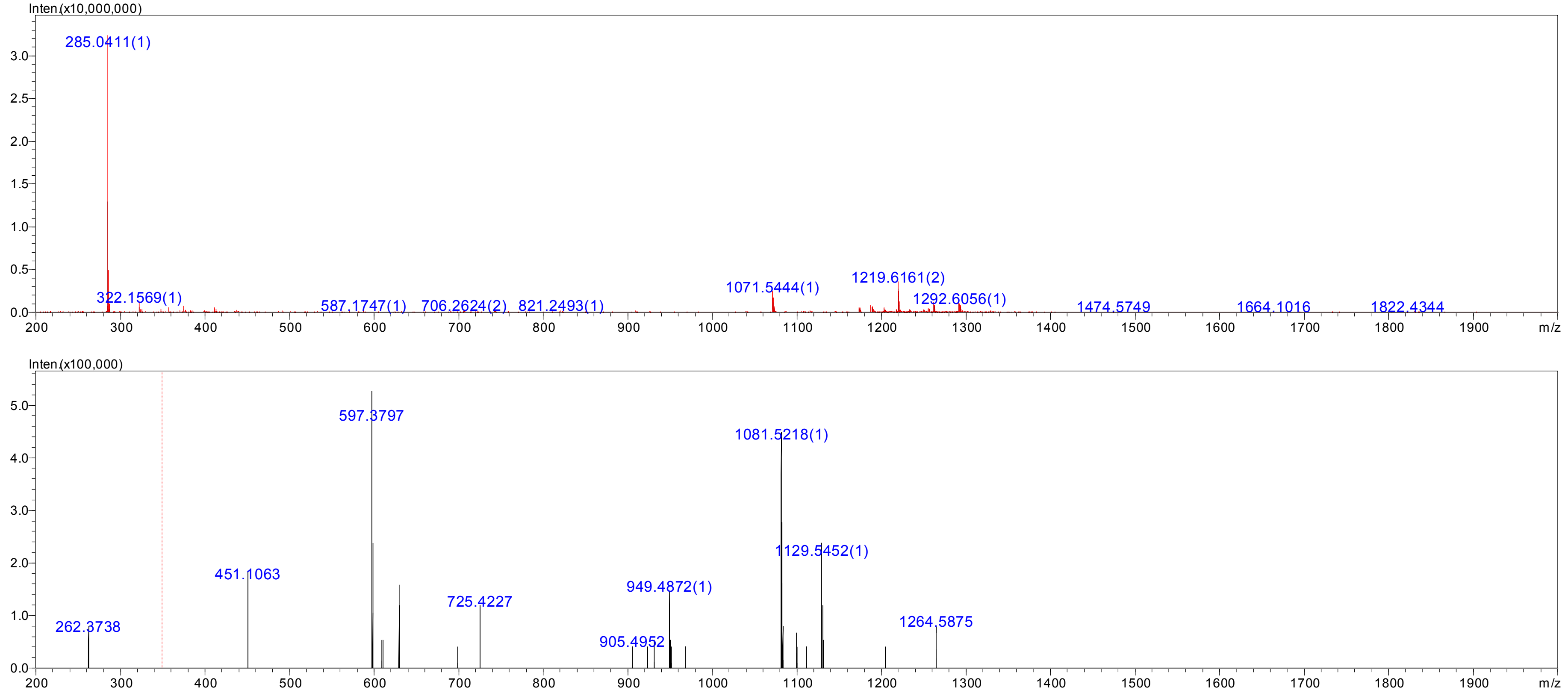

Peak 2 Retention time (min): 8.987

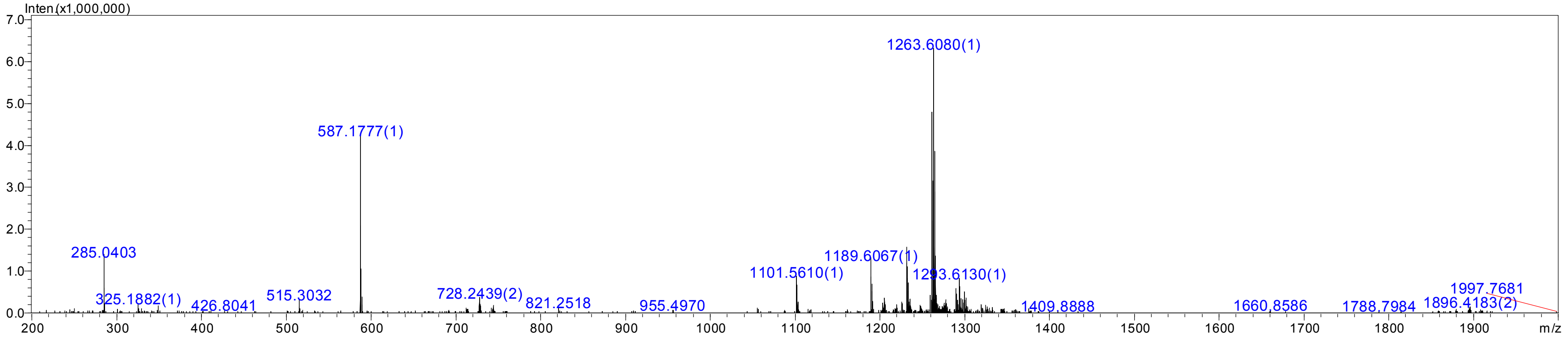

Peak 3 Retention time (min): 9.303

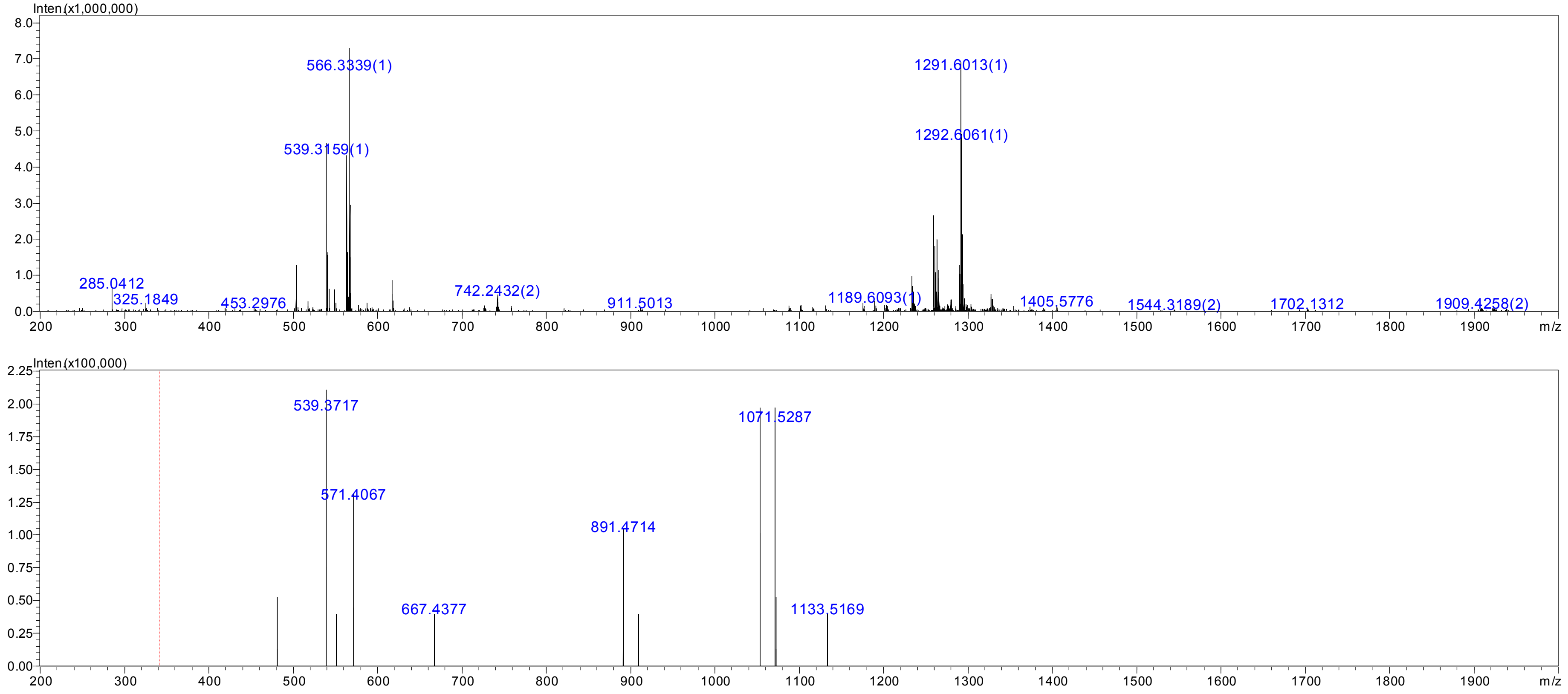

Peak 4 Retention time (min): 9.810

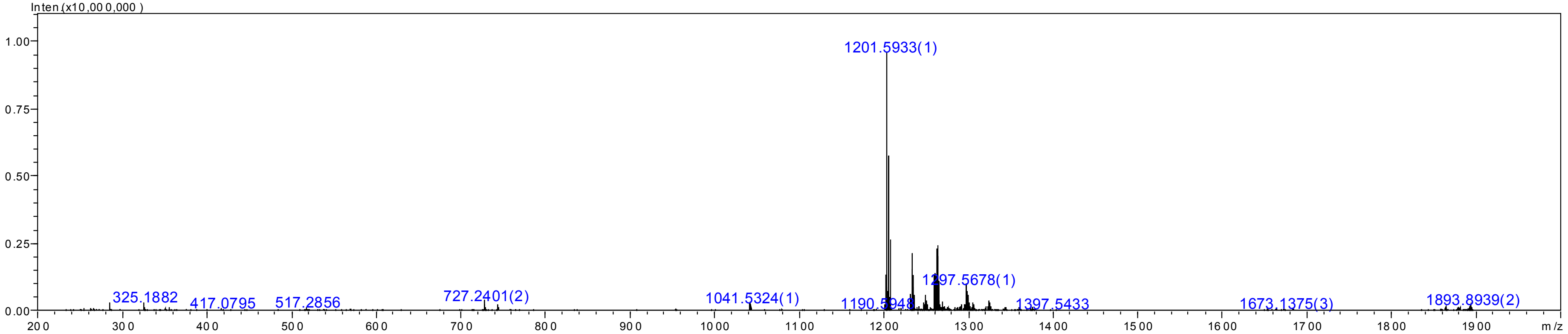

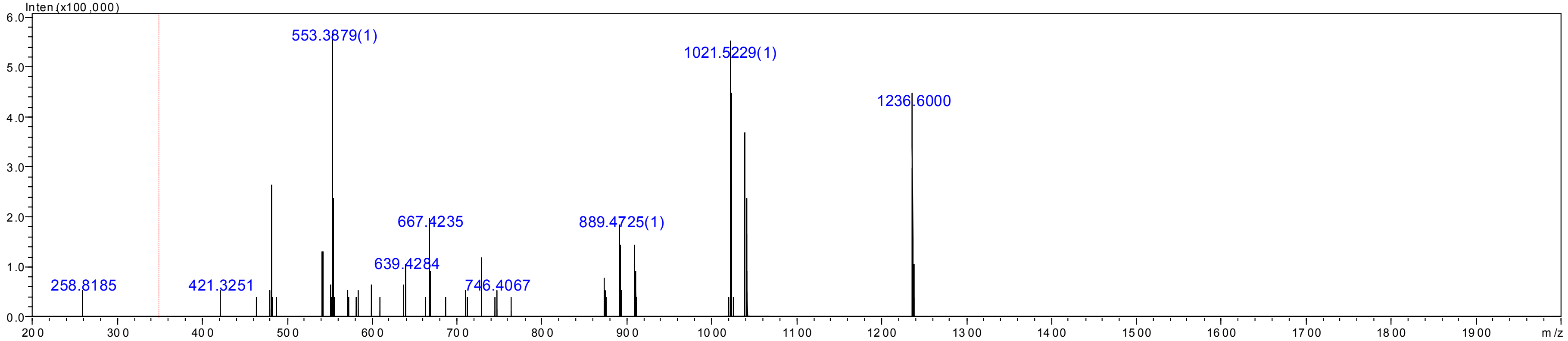

Peak 5 Retention time (min): 10.633

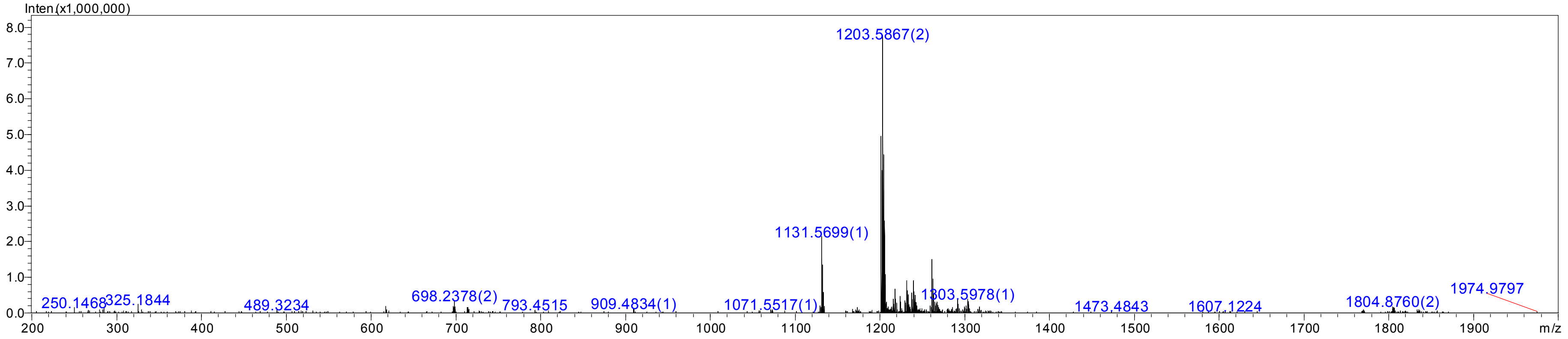

Peak 6   Retention time (min): 11.202

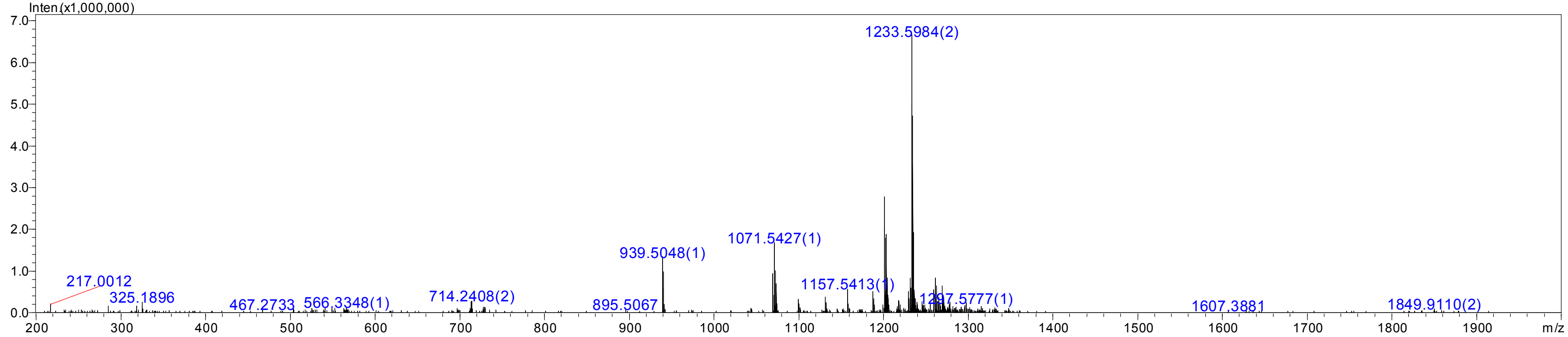

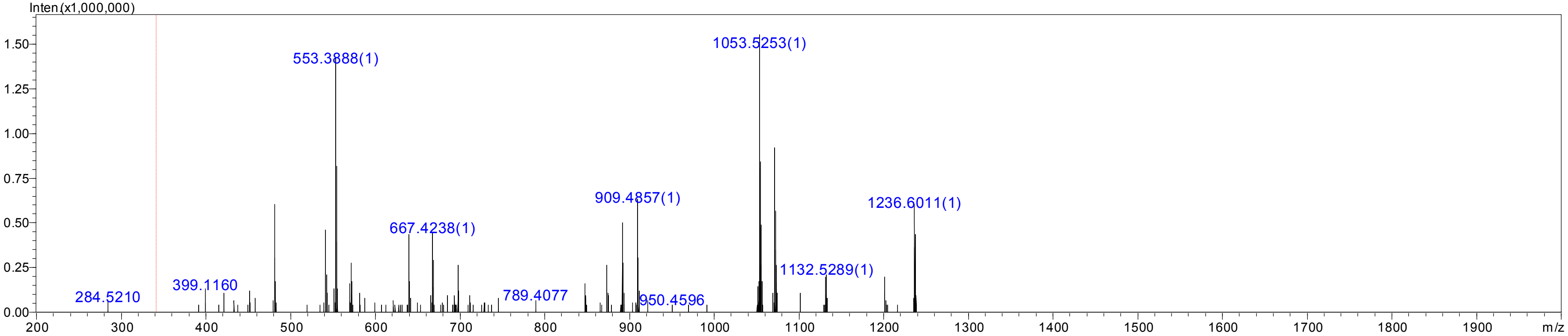

Peak 7 Retention time (min): 11.772

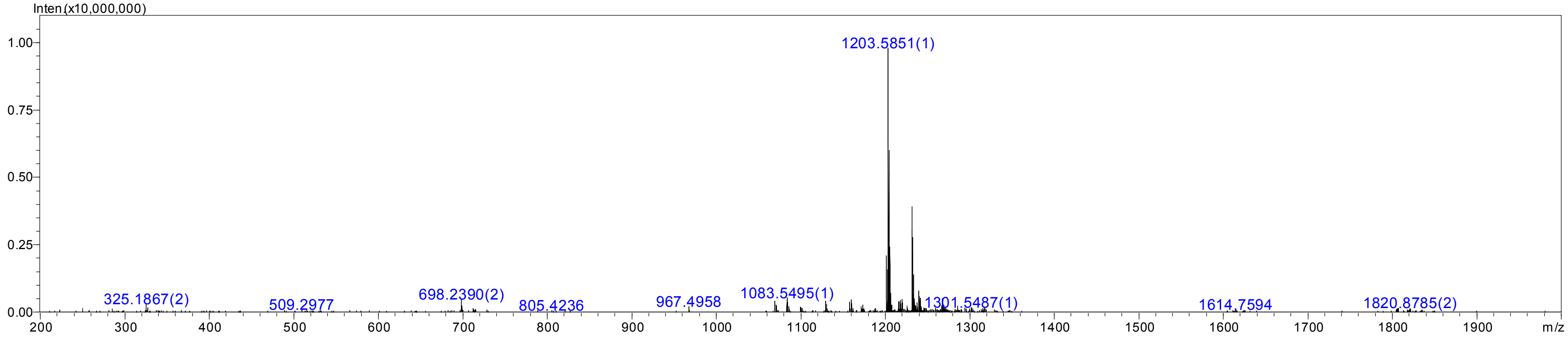

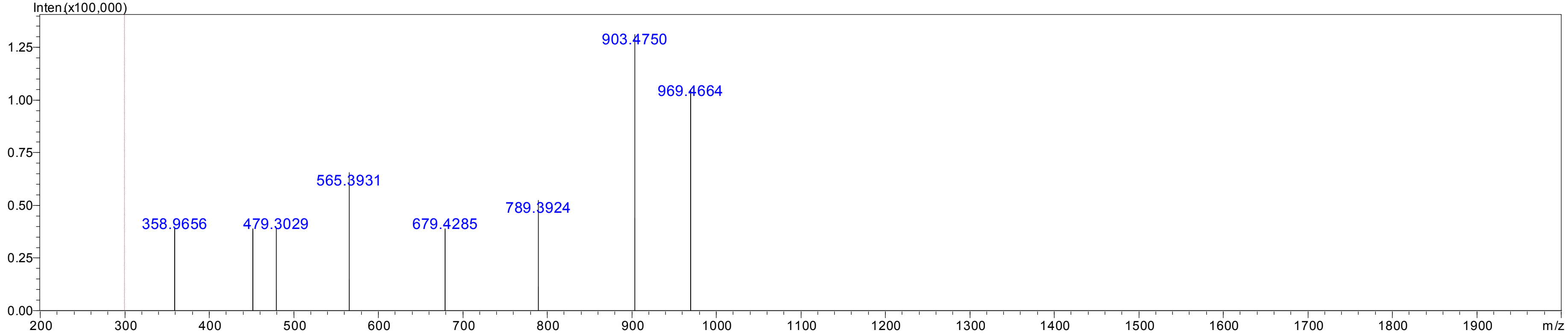

Peak 8 Retention time (min): 12.911

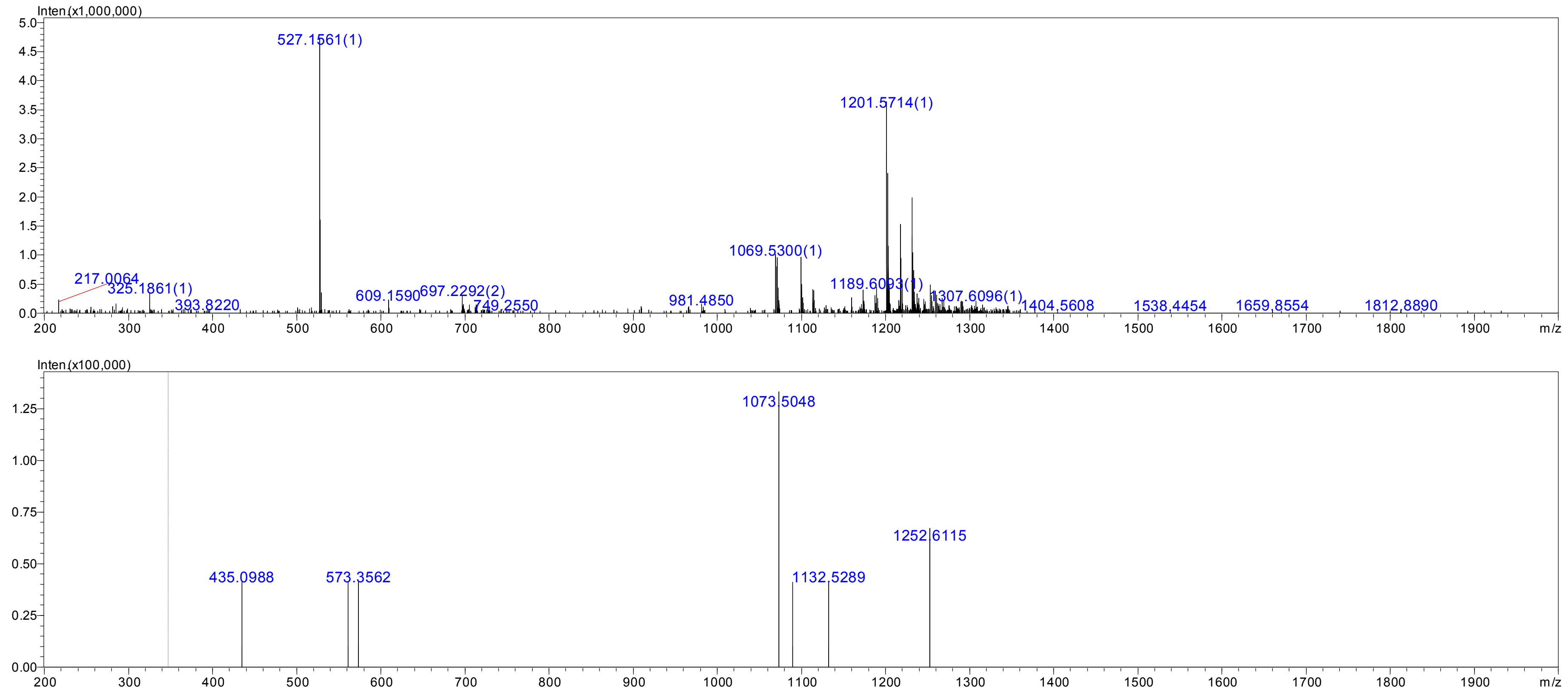

Peak9 Retention time (min): 13.544

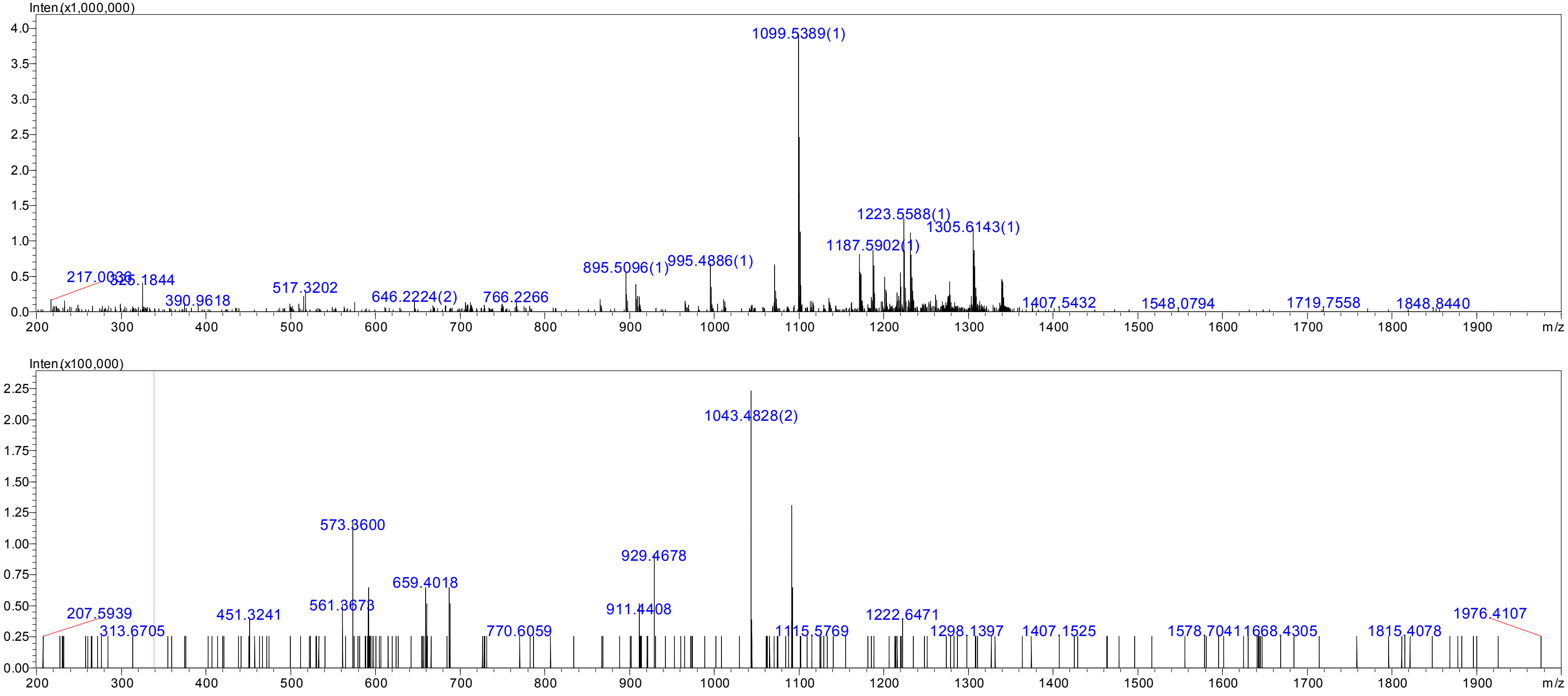

Peak 10   Retention time (min): 14.240

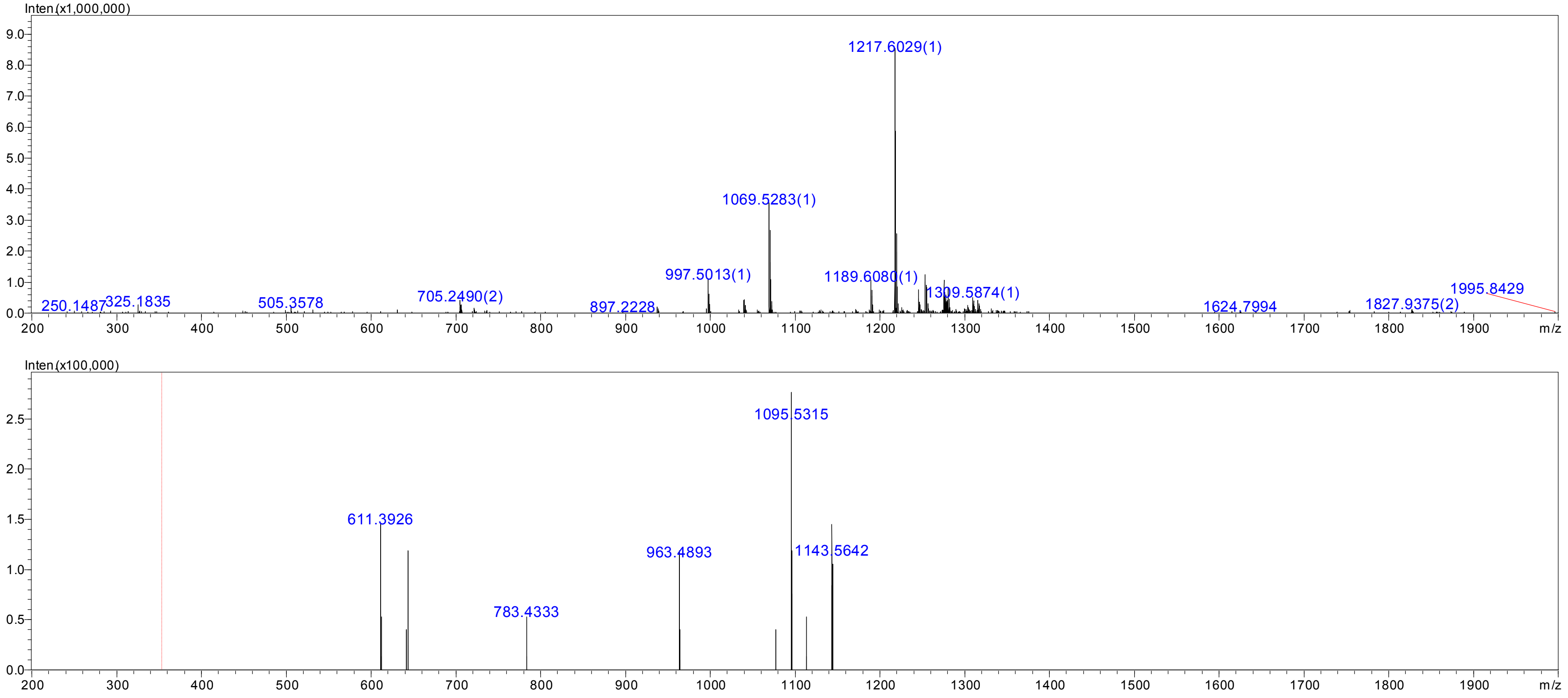

Peak 11 Retention time (min): 15.063

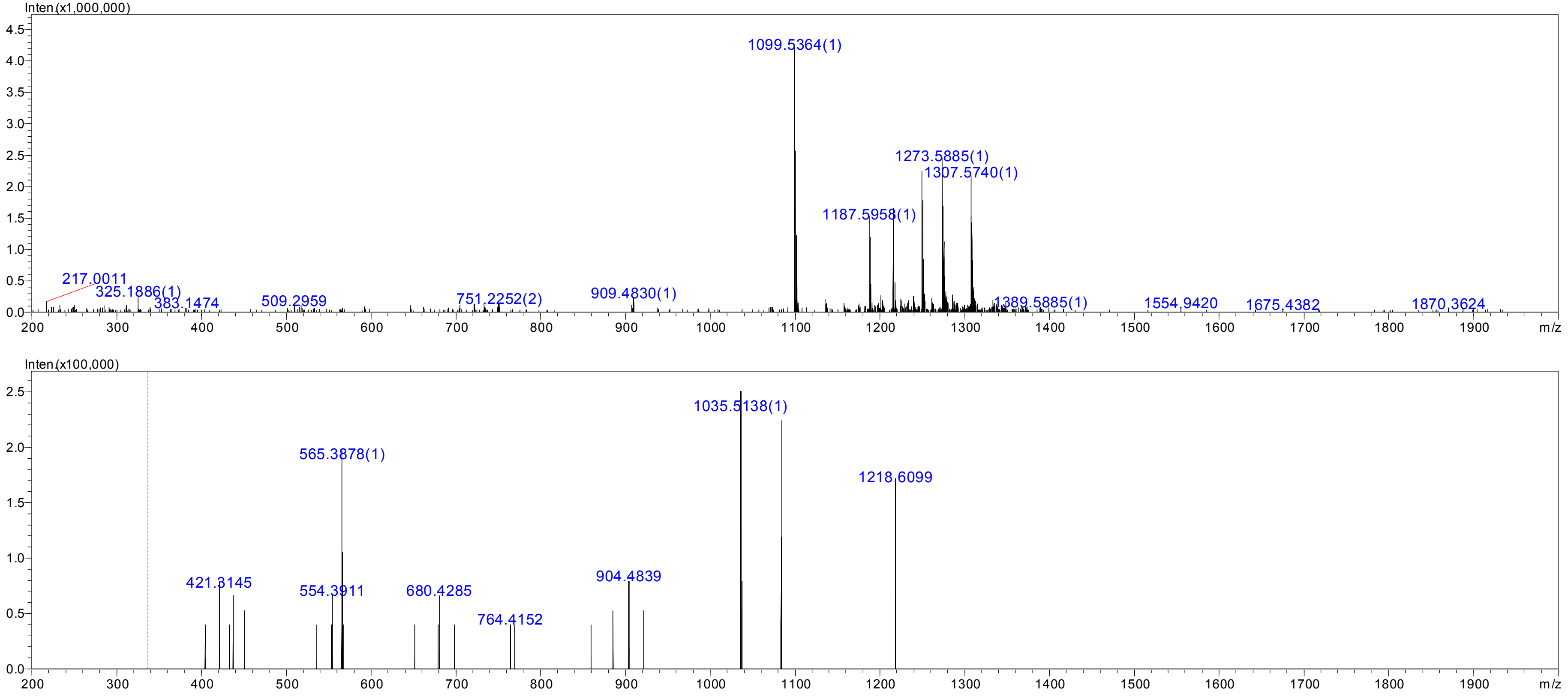

Peak 12 Retention time (min): 16.139

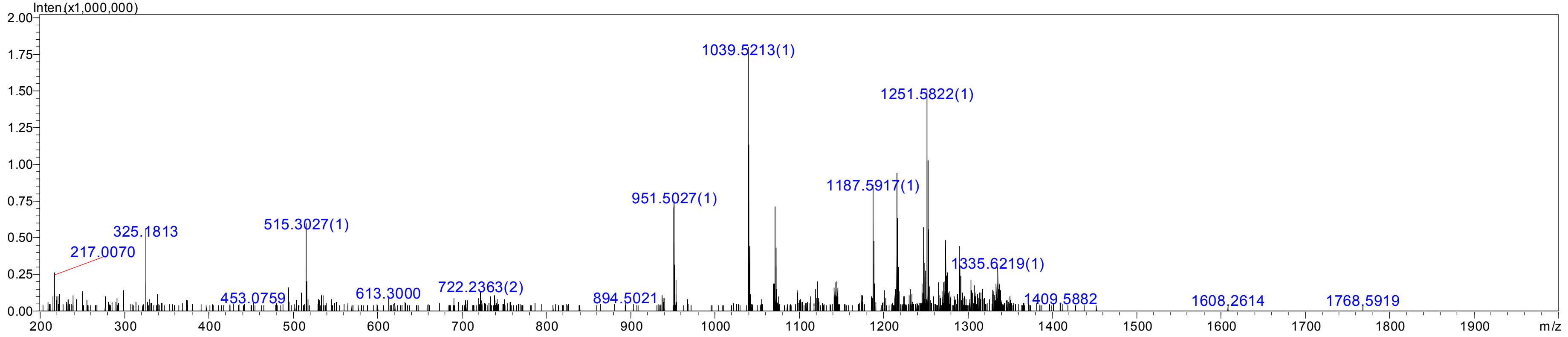

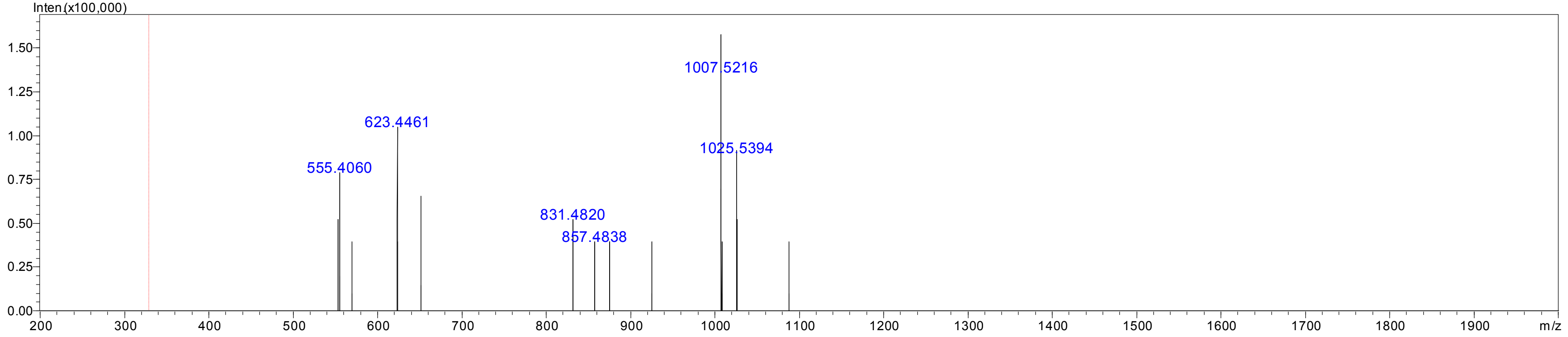

Peak 13 Retention time (min): 16.898

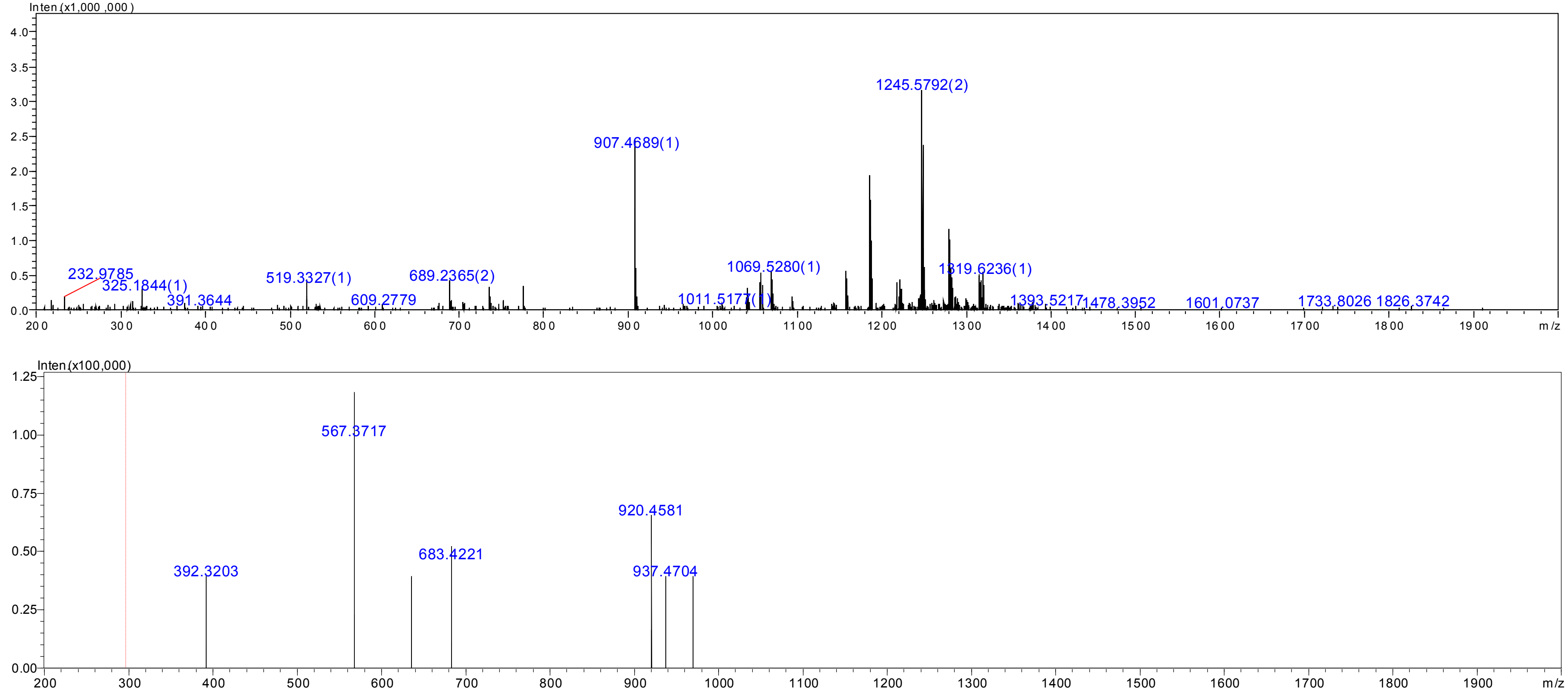

Peak 14 Retention time (min): 17.531

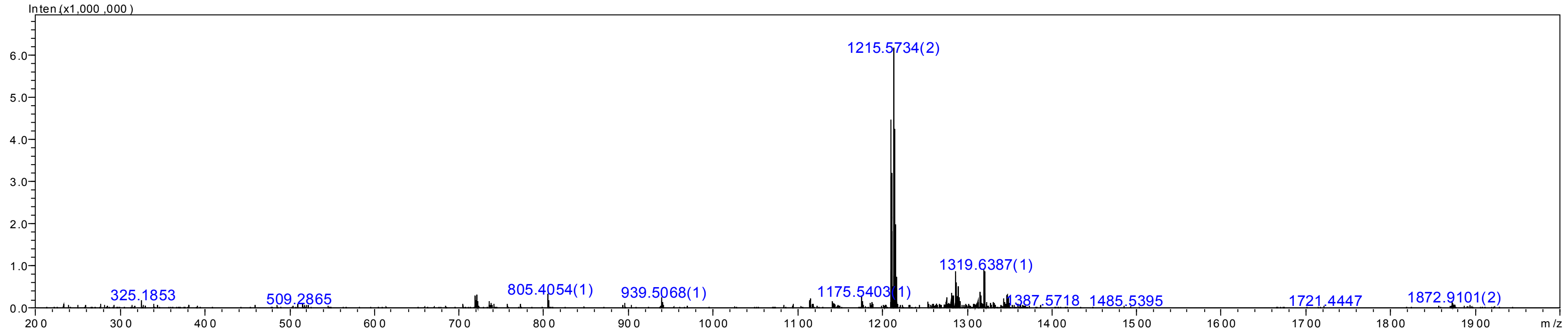

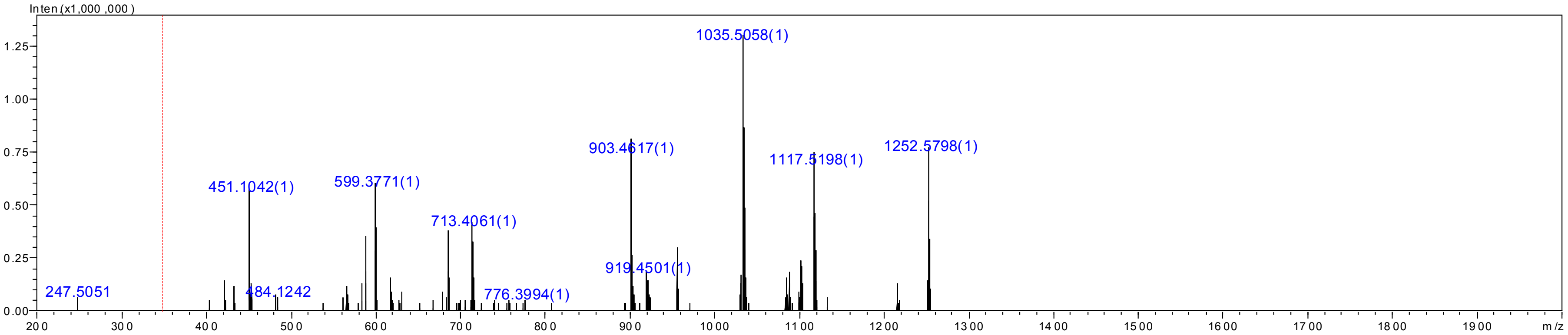

Peak 15 Retention time (min): 18.670

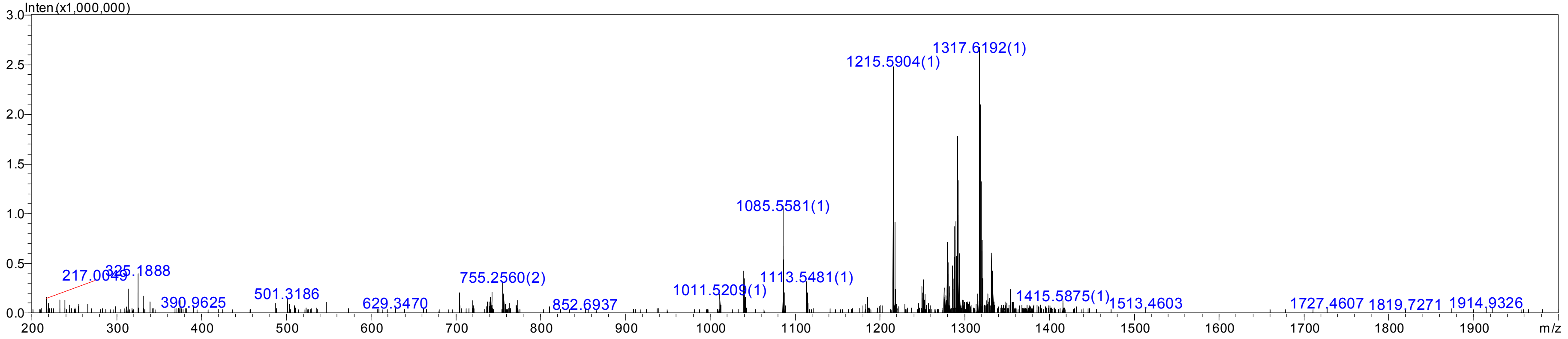

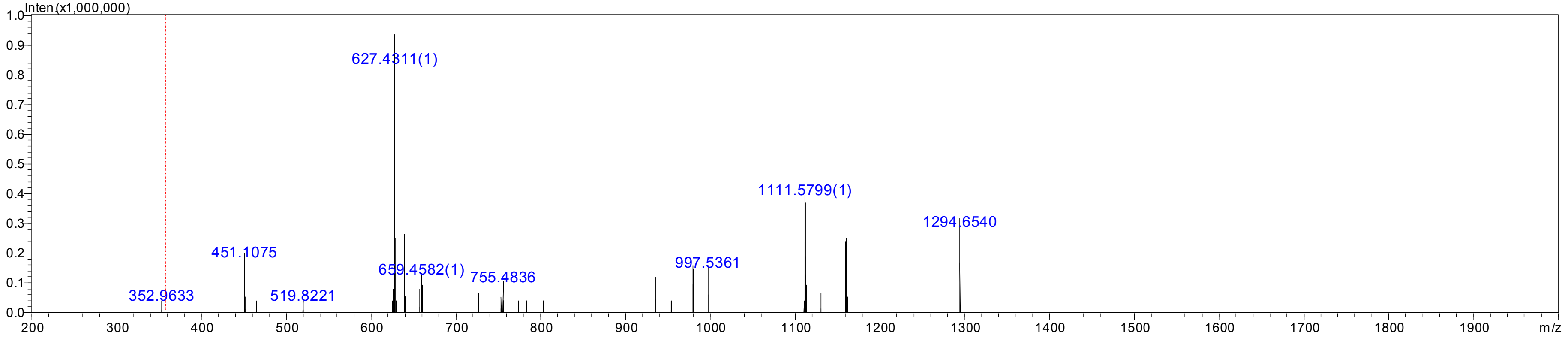

Peak 16 Retention time (min): 19.746

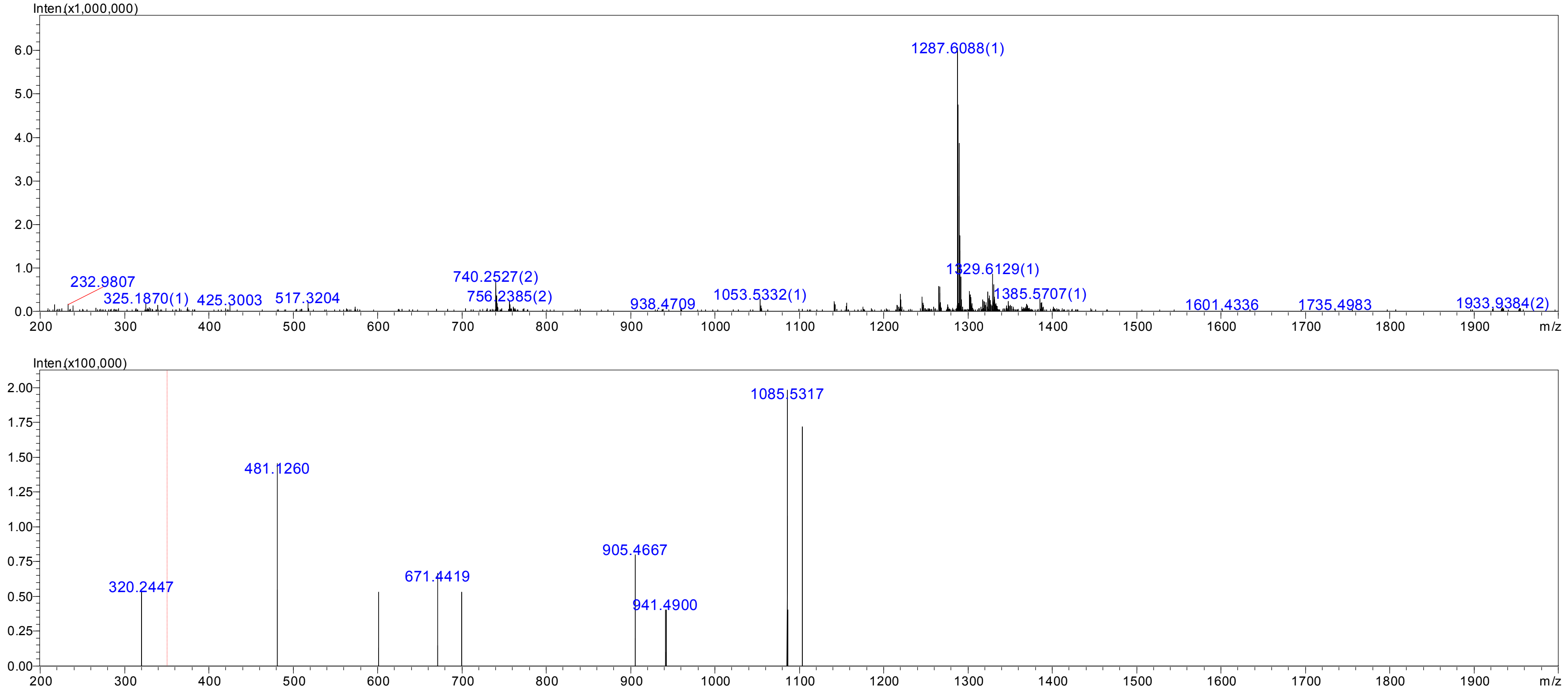

Peak 17 Retention time (min): 20.506

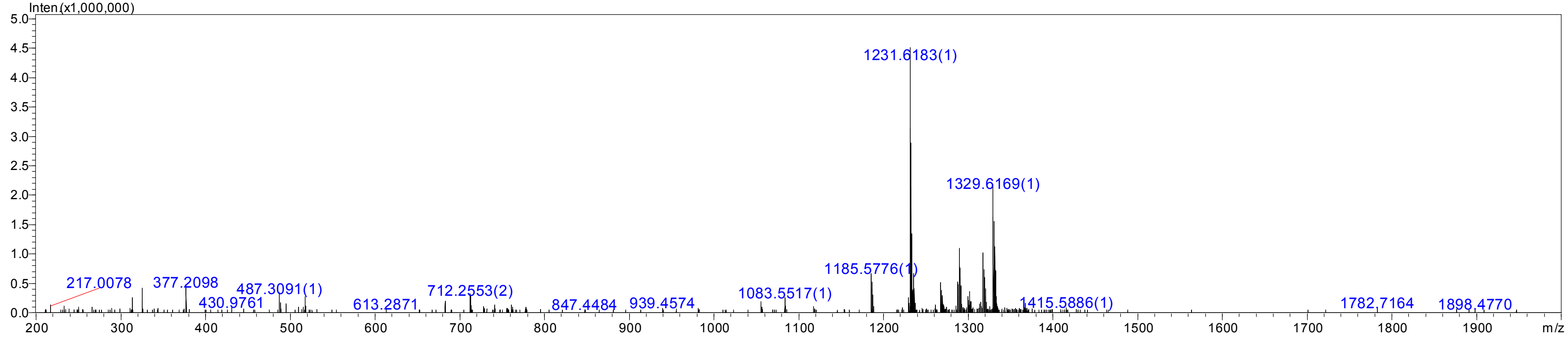

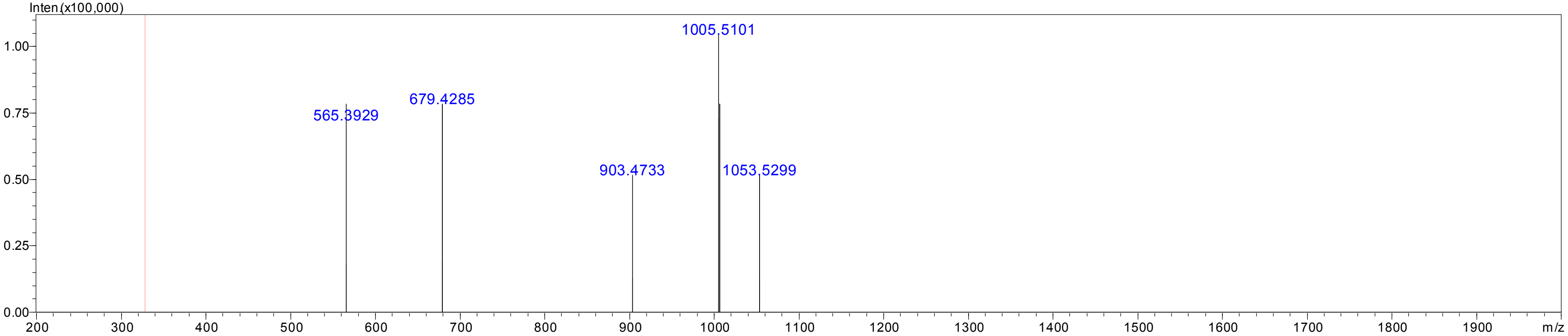

Peak 18 Retention time (min): 21.265

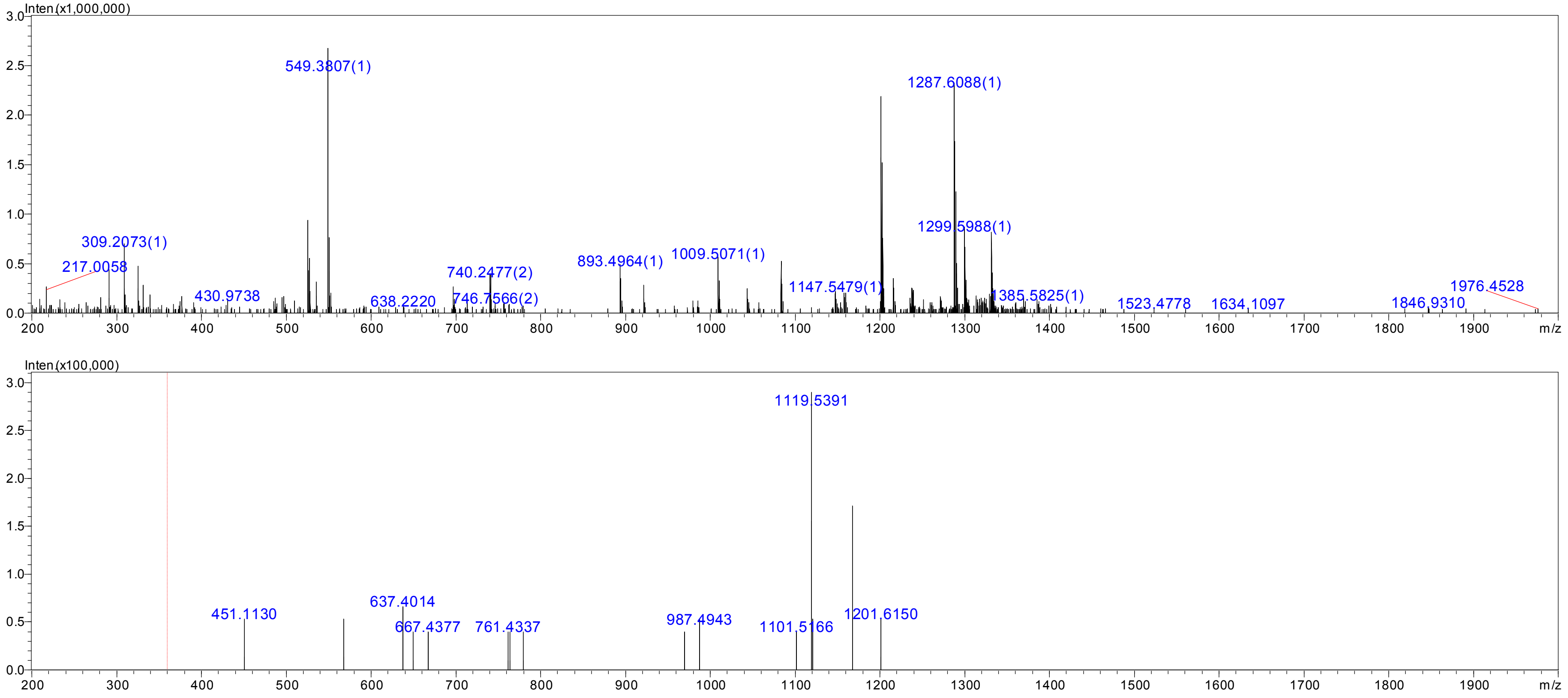

Peak 19 Retention time (min): 25.252

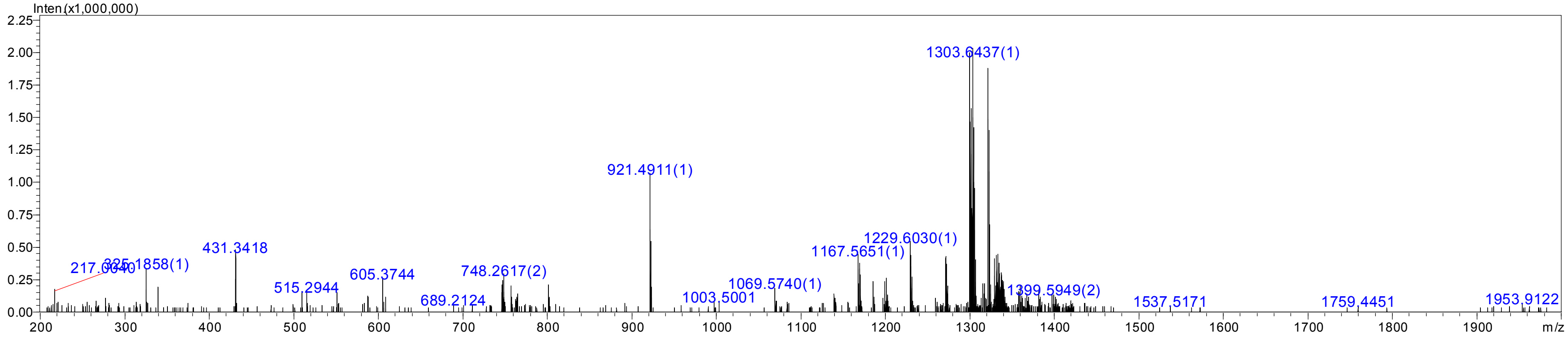

Peak 20 Retention time (min): 28.290

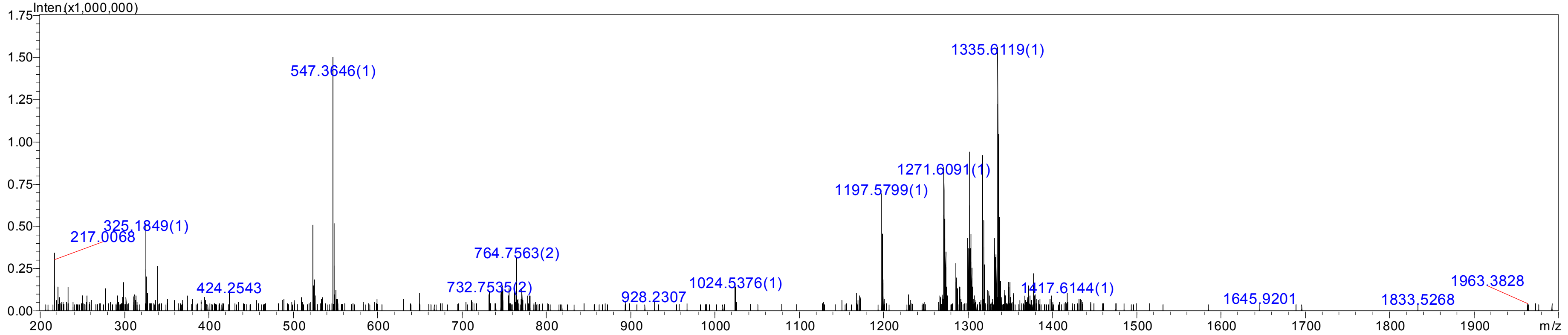

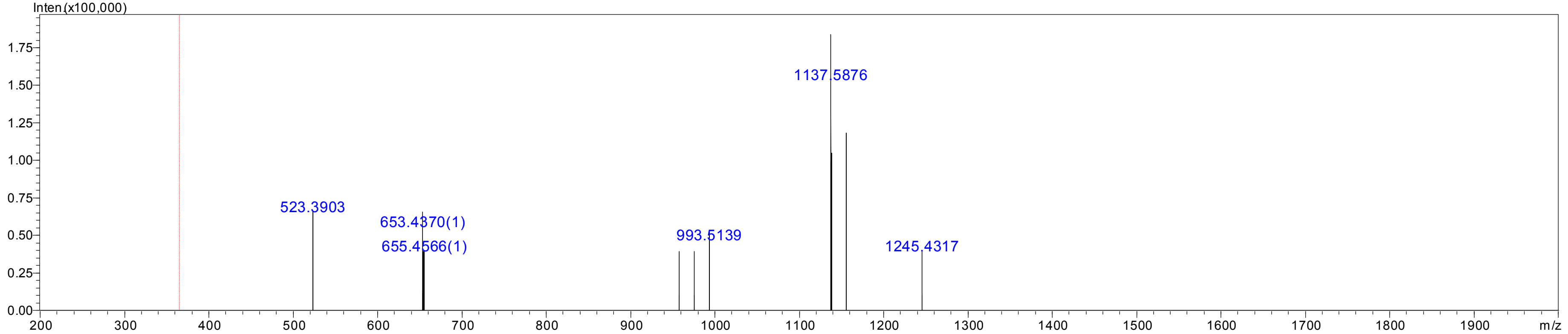

Peak 21 Retention time (min): 29.429

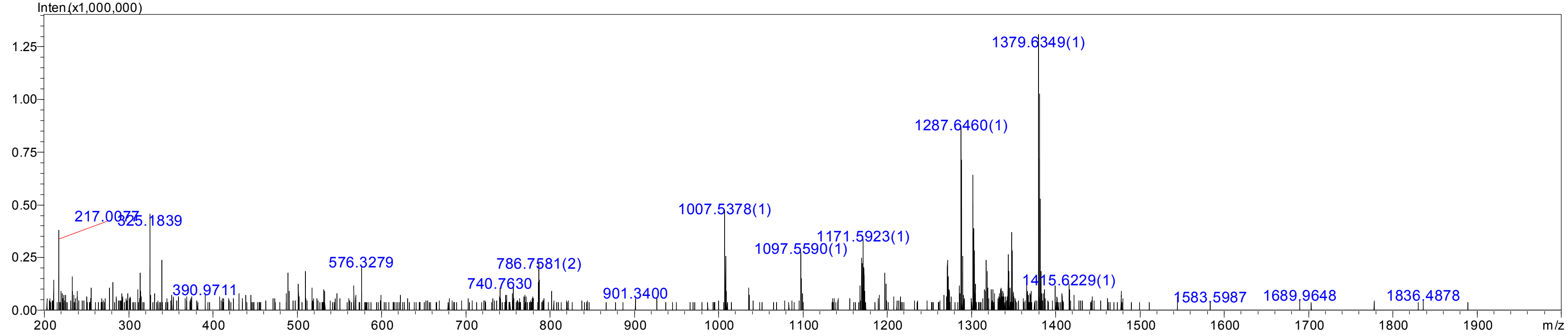

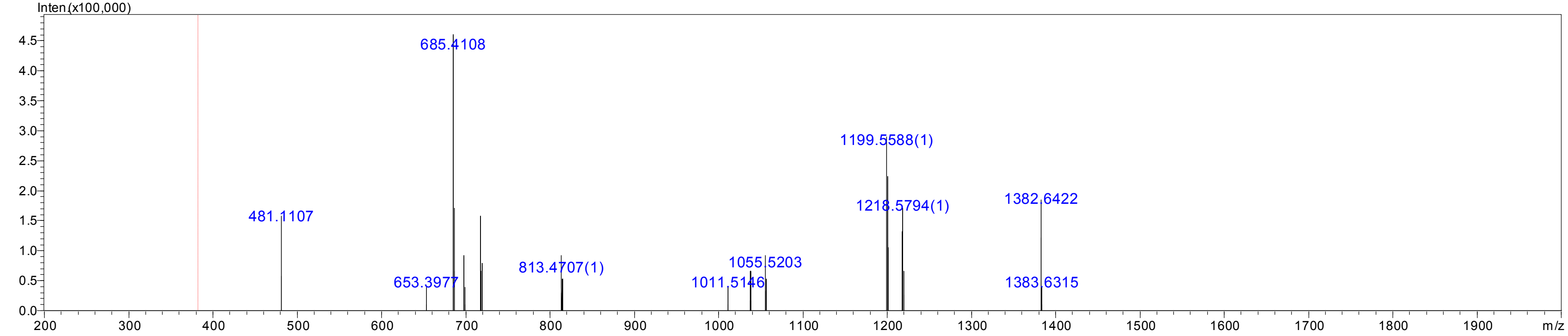

Peak 22 Retention time (min): 30.505

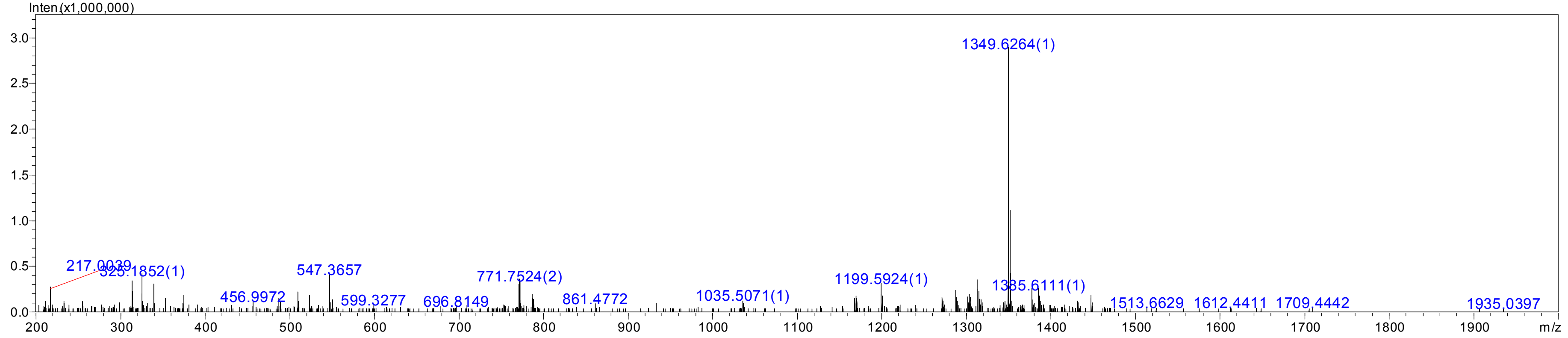

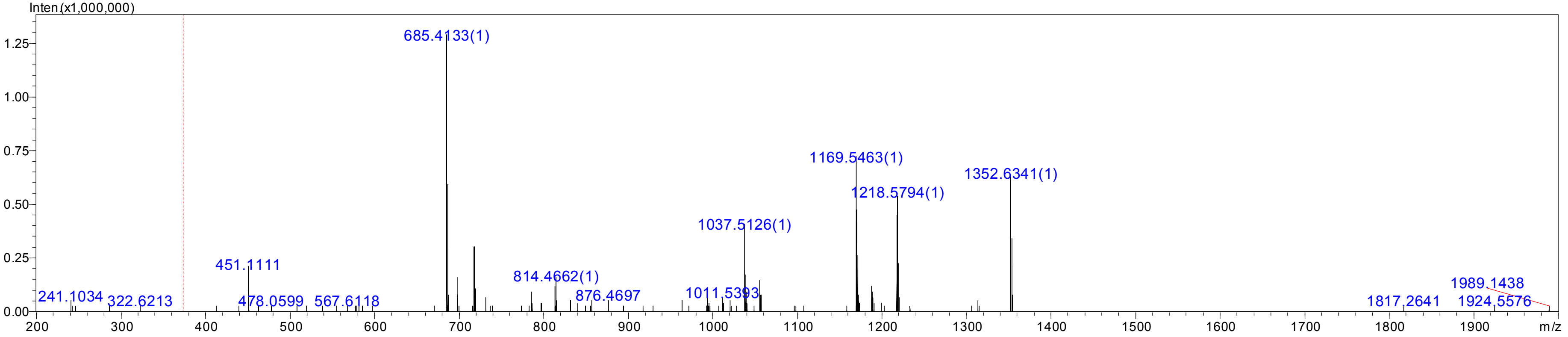

Peak 23   Retention time (min): 31.138

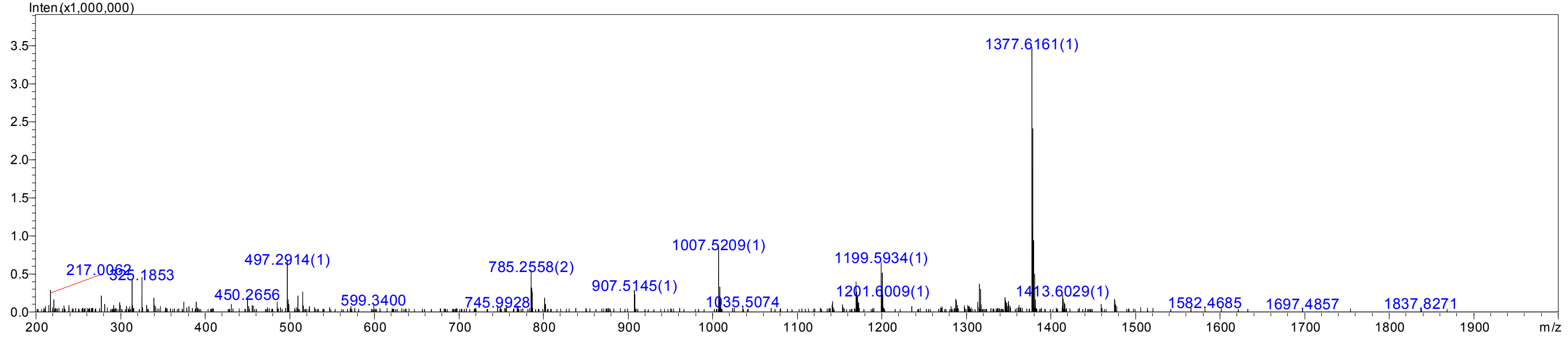

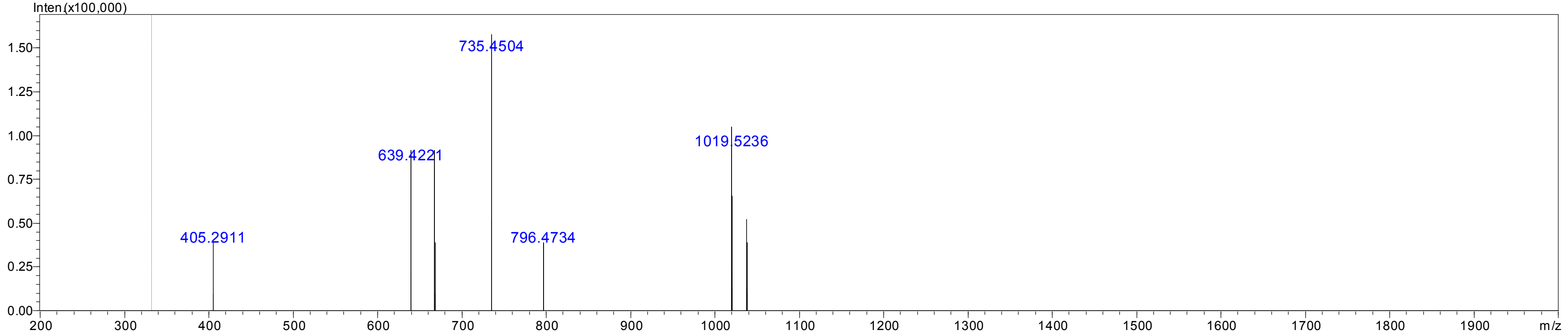

Peak 24 Retention time (min): 32.341

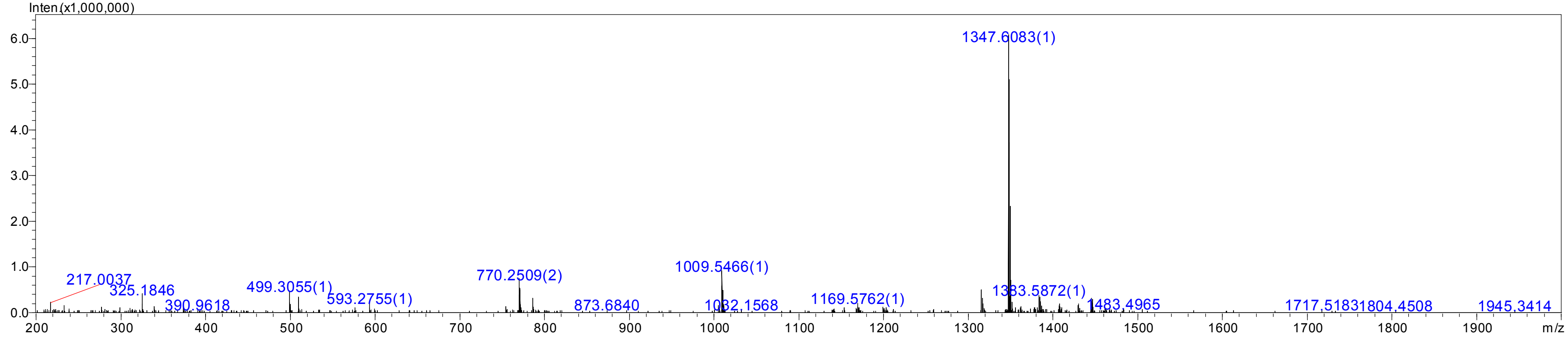

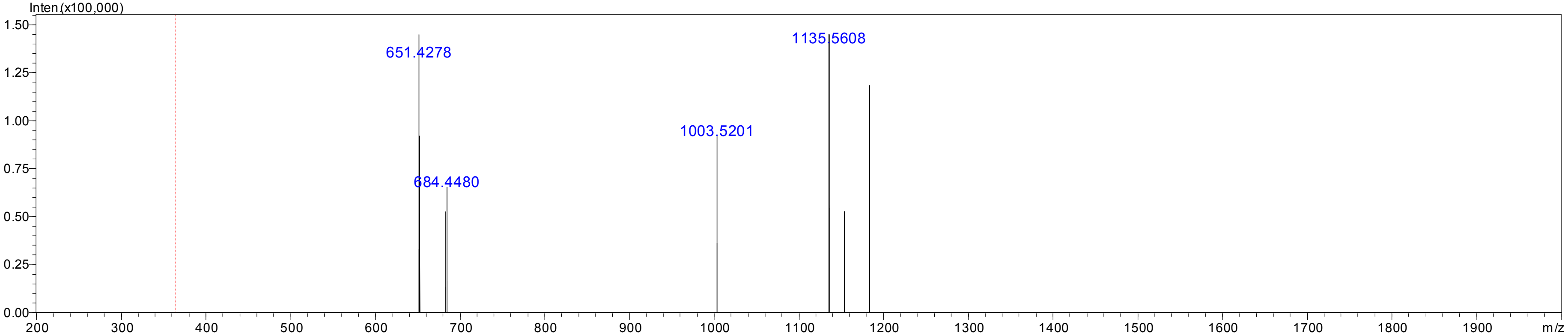

Peak 25 Retention time (min): 33.353

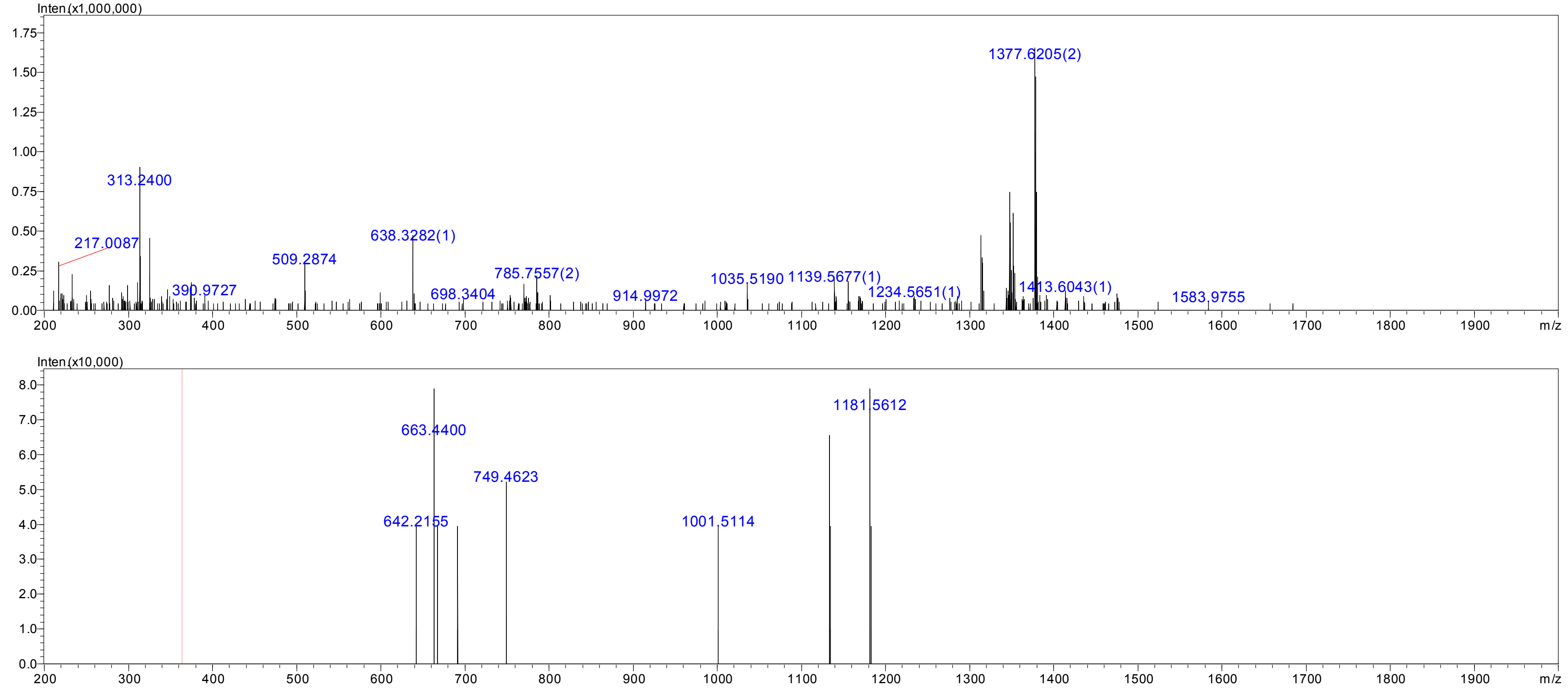

Peak 26 Retention time (min): 34.935

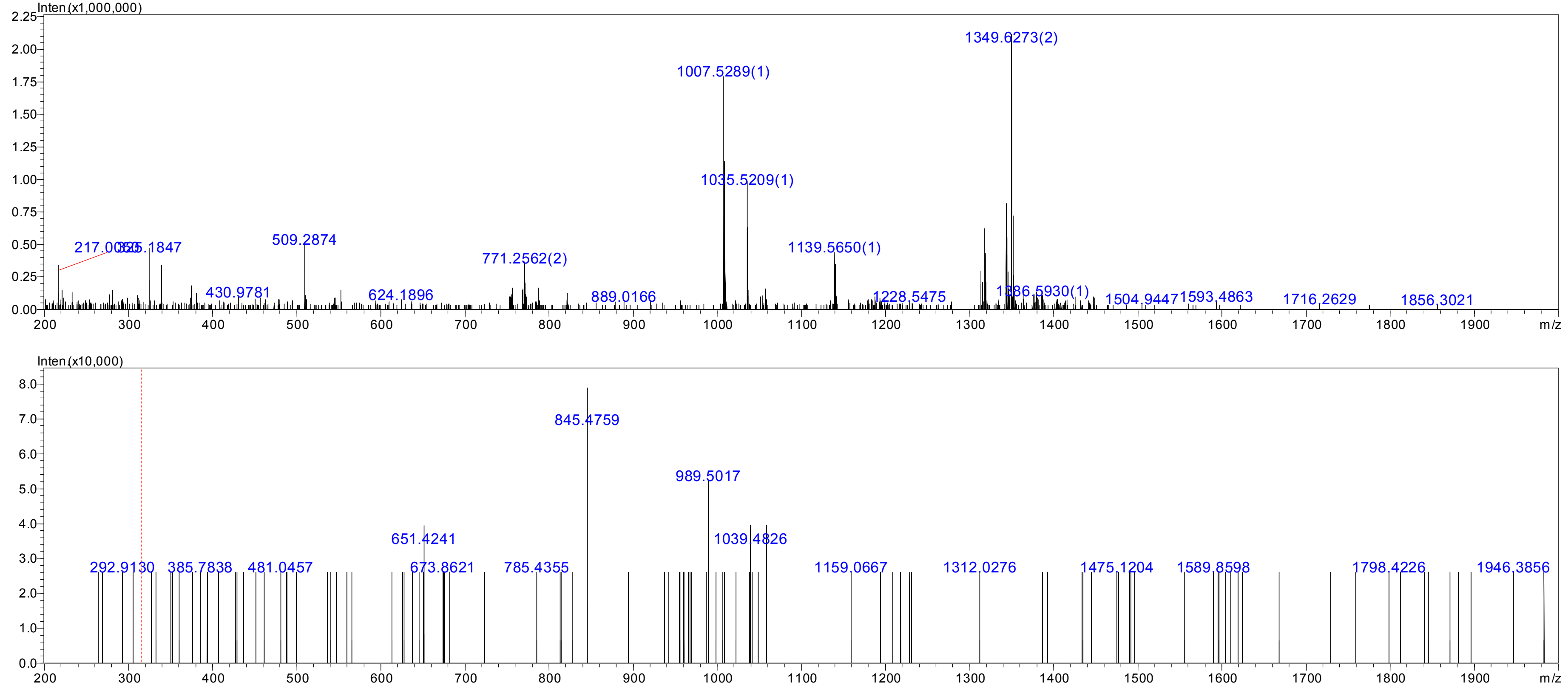

Peak 27 Retention time (min): 39.556

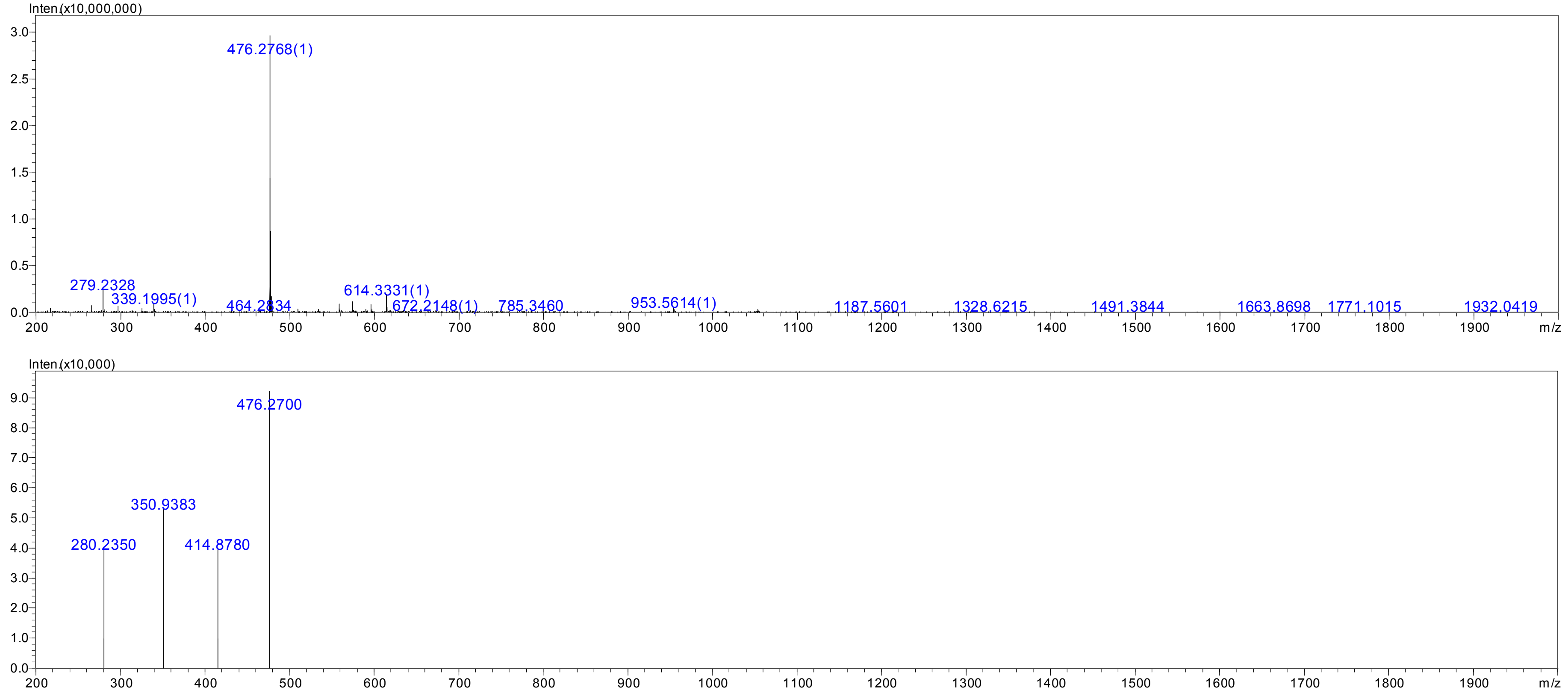

Peak 28　Retention time (min): 40.128

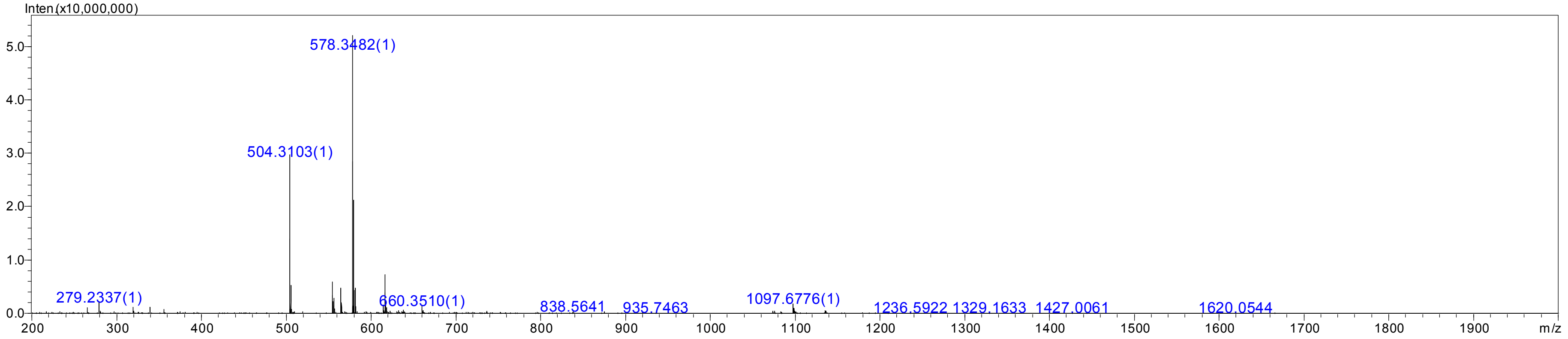

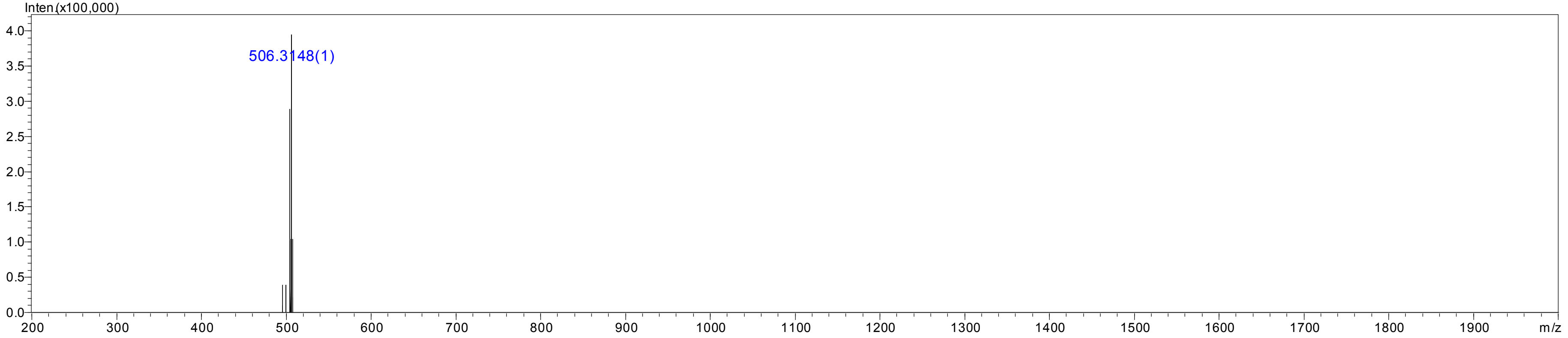

Peak 29 Retention time (min): 41.264

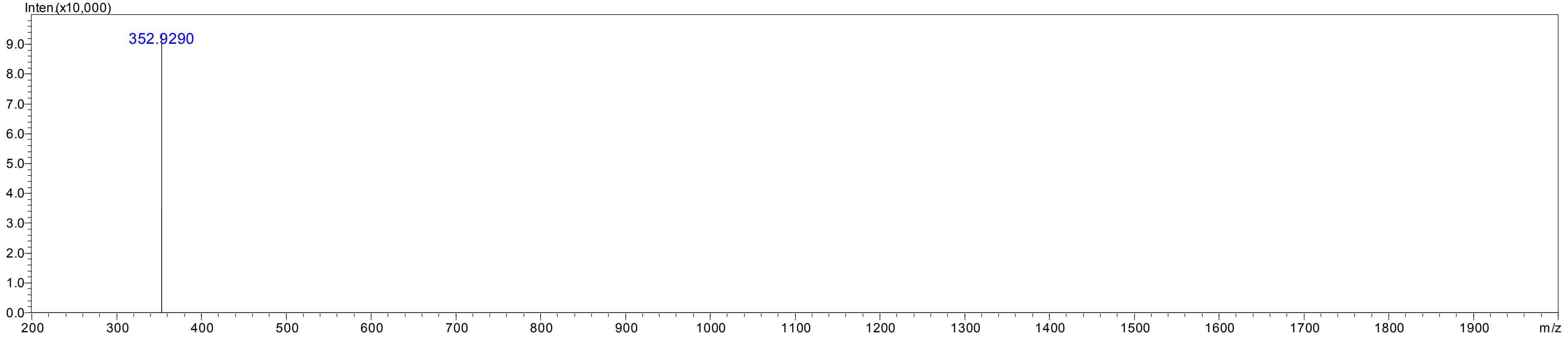

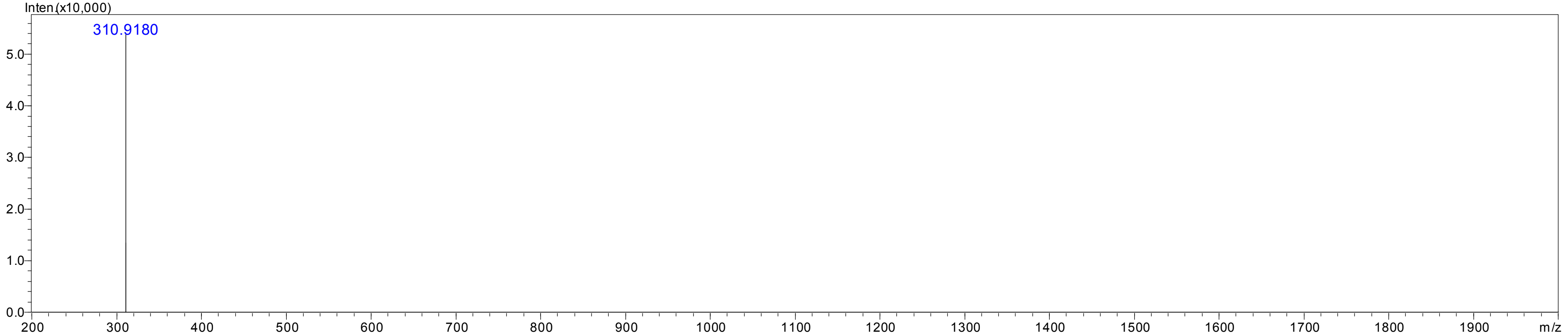

Peak 30 Retention time (min): 43.543

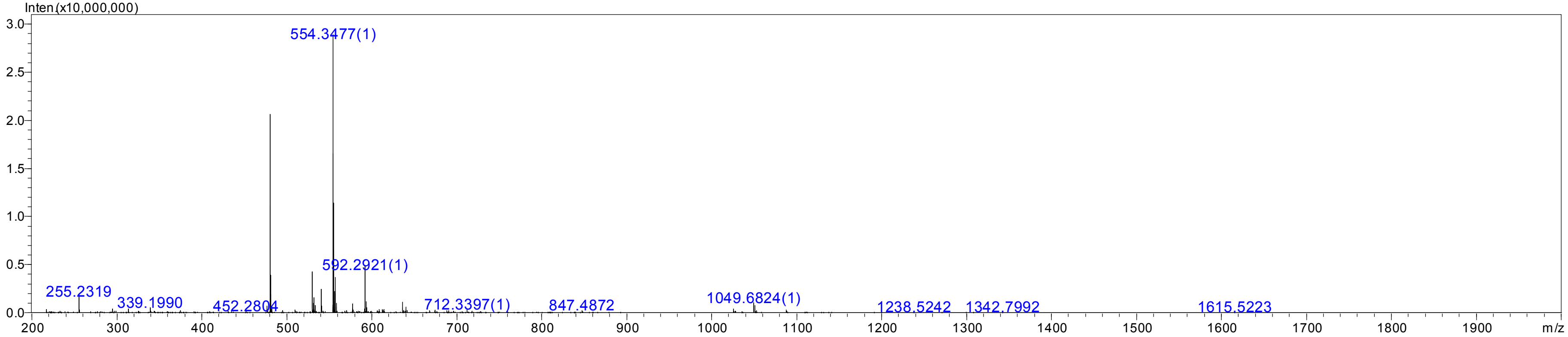

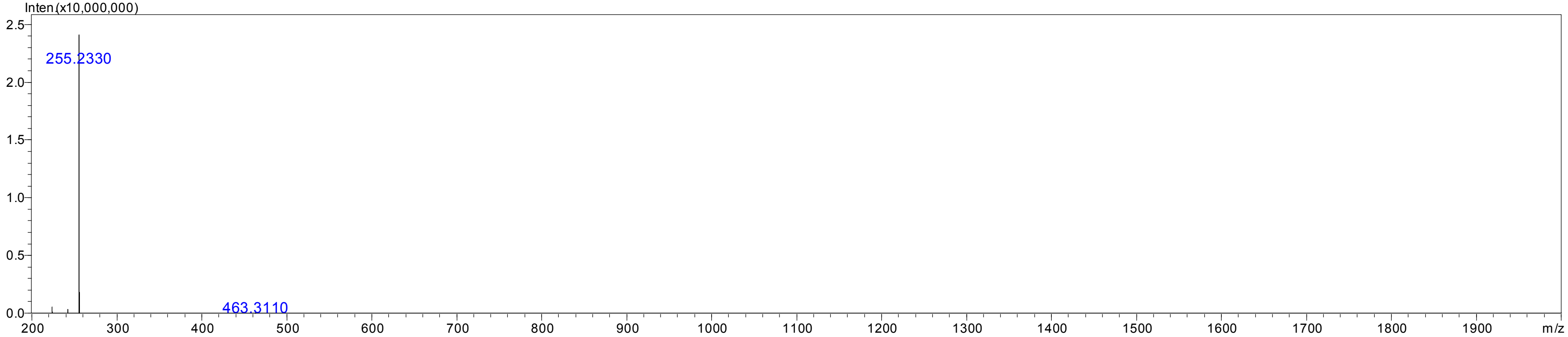

Peak 31　Retention time (min): 45.372

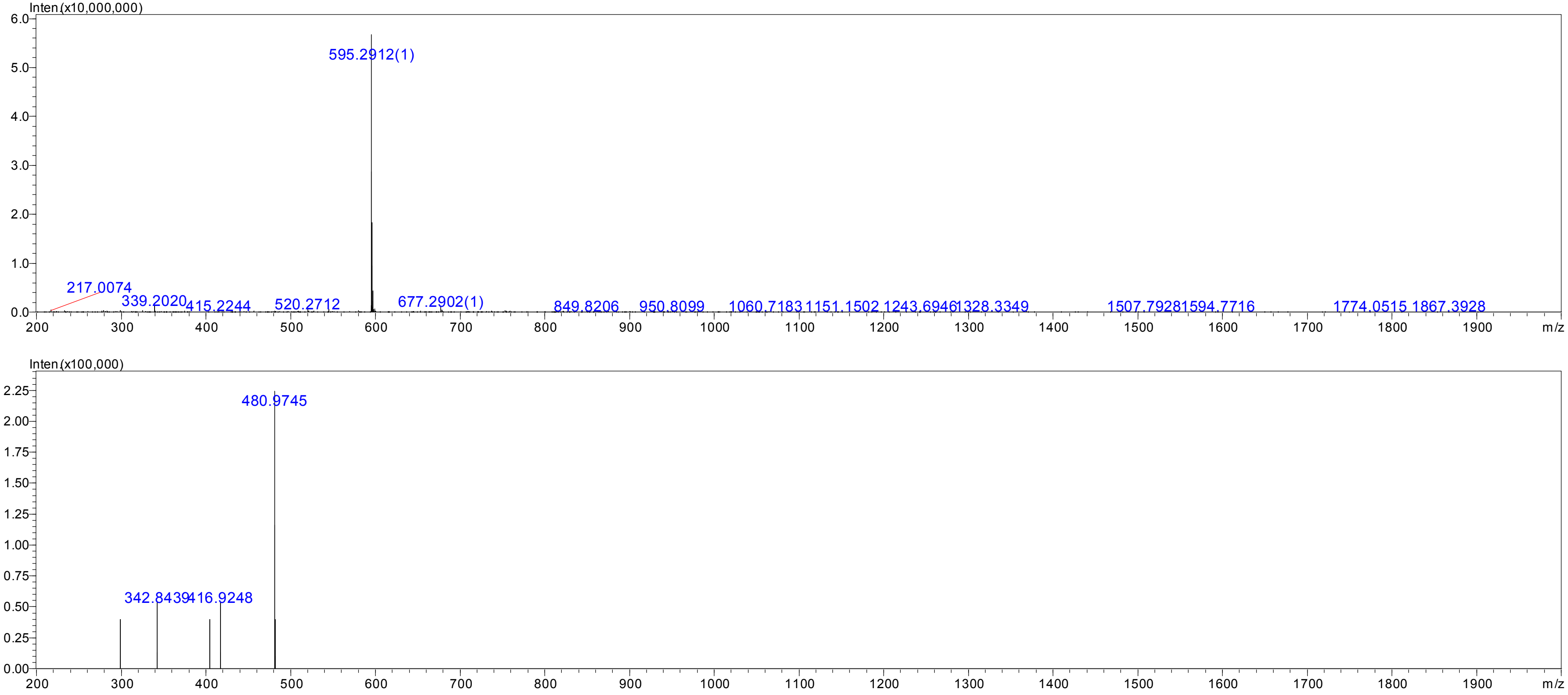

Peak 32　Retention time (min): 46.434

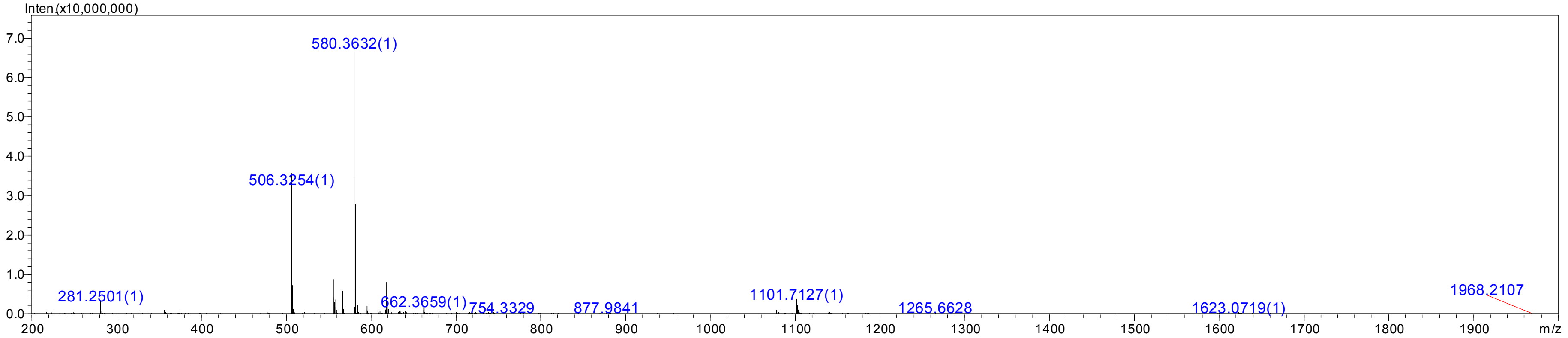

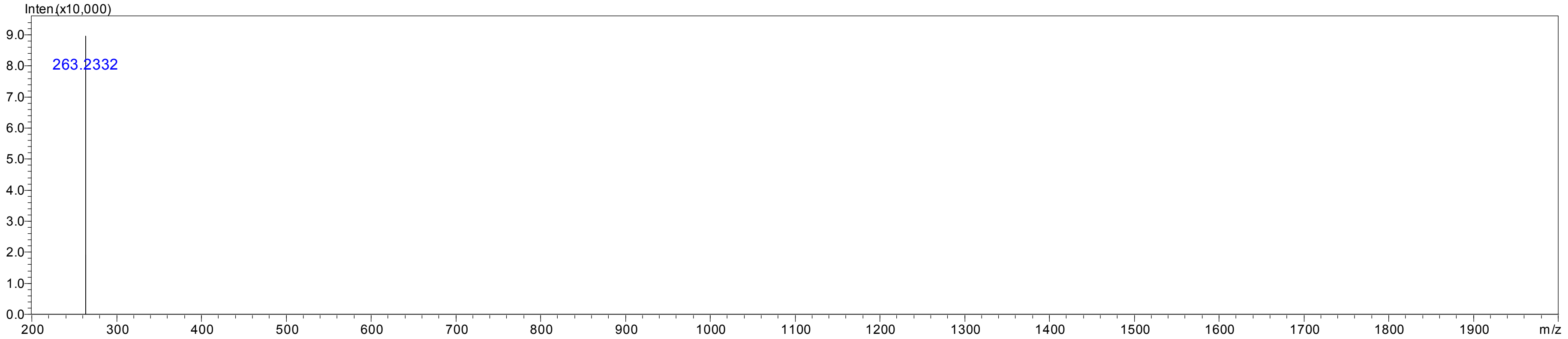

Peak 33 Retention time (min): 51.171

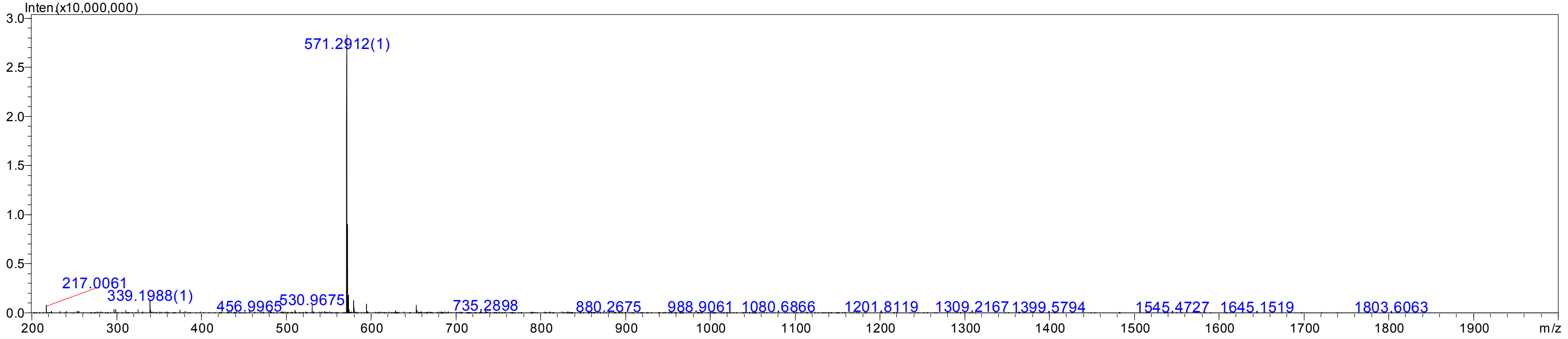

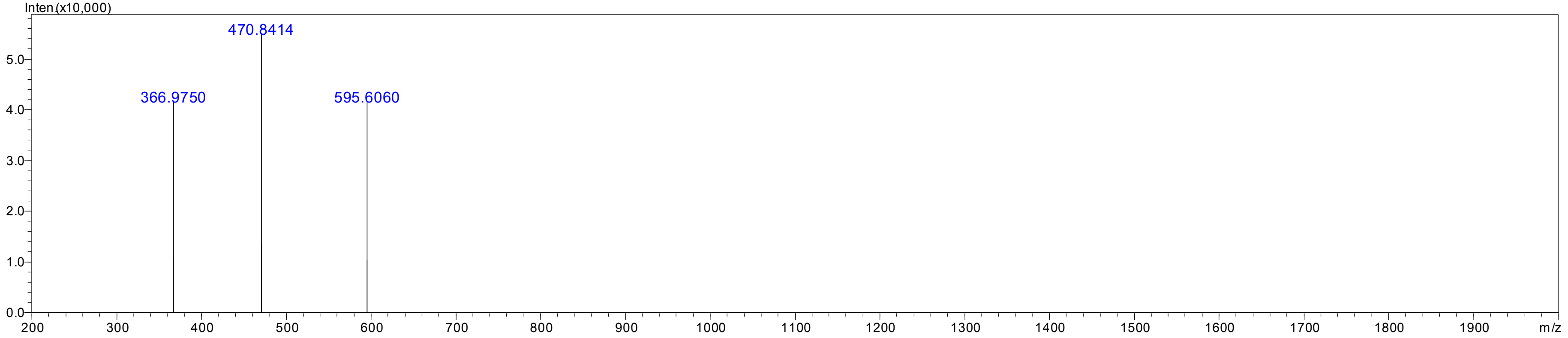

Supplement: Supplementary file 1 [file molecules-19-07568-s001.pdf]
